# Functional analysis of tumor-derived immunoglobulin lambda and its interacting proteins in cervical cancer

**DOI:** 10.1186/s12885-023-11426-9

**Published:** 2023-10-02

**Authors:** Juping Wang, Jiangni Huang, Hao Ding, Jing Ma, Haohua Zhong, Fanlu Wang, Yupeng Chen, Hui Peng

**Affiliations:** 1 Department of Pathology, The First Affiliated Hospital of Fujian Medical University, Fuzhou, China; 2 Department of Pathology, Binhai Branch of National Regional Medical Center, The First Affiliated Hospital, Fujian Medical University, Fuzhou, China; 3 Department of Pathophysiology, School of Basic Medical Sciences, Youjiang Medical University for Nationalities, Baise, China; 4 Fujian Provincial Institutes of Brain Disorders and Brain Sciences, The First Affiliated Hospital, Fujian Medical University, Fuzhou, China; 5 Department of Neurosurgery, The First Affiliated Hospital, Neurosurgery Research Institute, Fujian Medical University, Fuzhou, China

**Keywords:** Immunoglobulin lambda, Cervical cancer, Function, Analysis, Bioinformatics

## Abstract

**Background:**

Immunoglobulin lambda (Igλ) has been reported to be expressed in many normal and tumor tissues and cells. However, the function and clinical significance of tumor-derived Igλ remain unclear.

**Methods:**

The differential expressions of Immunoglobulin Lambda Constants (IGLCs) in cervical squamous cell carcinoma and endocervical adenocarcinoma (CESC) were examined with The Cancer Genome Atlas (TCGA), Genotype-Tissue Expression (GTEx), and Human Protein Atlas (HPA) databases. The effects of IGLCs on patient clinical phenotypes and prognosis were explored via bioinformatics analyses based on the TCGA databases. We used the bioinformatics analyses based on the TCGA and GTEx databases to elucidate the correlations among IGLC expressions, immunomodulator expressions, tumor stemness, and infiltration scores of tumor infiltrating immune cells. Co-immunoprecipitation (Co-IP) and silver staining combined with liquid chromatography-tandem mass spectrometry (LC–MS/MS) were used to obtain potential tumor-derived Igλ-interacting proteins. Functional annotation of candidate proteins identified by LC–MS/MS was performed in Database for Annotation, Visualization and Integrated Discovery (DAVID). The bioinformatics analyses of 7 IGLCs in CESC and normal cervical tissues were performed based on TCGA, GTEx, and Gene Expression Profiling Interactive Analysis 2 (GEPIA2) databases. Protein–protein interaction (PPI) network was analyzed based on tumor-derived Igλ-interacting proteins in Search Tool for the Retrieval of Interacting Genes/Proteins (STRING) database. Immunohistochemistry (IHC) was used to validate the expressions of IGLCs in CESC.

**Results:**

We found that the expressions of the majority of IGLCs (IGLC1, IGLC2, IGLC3, IGLC4, IGLC5, IGLC6, and IGLC7) were upregulated in CESC tissues, compared with those in normal cervical tissues. The expressions of IGLC5 and IGLC7 had significant difference in different pathologic metastasis (M), one of tumor, node, and metastasis (TNM) staging system, categories of CESC. Except for disease-free interval (DFI), 4 IGLC (IGLC1, IGLC2, IGLC3, and IGLC7) expression levels were positively associated with patient overall survival (OS), disease-specific survival (DSS), and progression-free interval (PFI) respectively in CESC tissues. 5 IGLC (IGLC1, IGLC2, IGLC3, IGLC6, and IGLC7) expressions were positively correlated with the expressions of a majority of immunomodulators respectively in CESC tissues. Tumor stemness was negatively correlated with the expressions of 4 IGLCs (IGLC1, IGLC2, IGLC3, and IGLC7) respectively in CESC tissues. Except for IGLC4, IGLC5, and IGLC7, 4 IGLC (IGLC1, IGLC2, IGLC3, and IGLC6) expressions were positively correlated with infiltration scores of 6 tumor-infiltrating immune cells (B cell, T cell CD4, T cell CD8, neutrophil, macrophage, and DC). After analyses of the above bioinformatics data of tumor-derived Igλ, Co-IP and LC–MS/MS were used to confirm that 4 proteins (RPL7, RPS3, H1-5, and H1-6) might interact with tumor-derived Igλ in cervical cancer cells. Functional analyses of these candidate proteins showed that they interacted with many proteins and were involved in various cellular biological processes. Finally, IHC was used to further confirm the above bioinformatics results, it was indicated that the expression level of Igλ in cervical adenocarcinoma and cervical squamous cell carcinoma was higher than that in normal cervical tissue.

**Conclusion:**

This study comprehensively investigated the functions of tumor-derived Igλ and its interacting proteins based on bioinformatics analysis and the potential value of Igλ as a prognostic and therapeutic marker for CESC, providing new direction and evidence for CESC therapy.

**Supplementary Information:**

The online version contains supplementary material available at 10.1186/s12885-023-11426-9.

## Introduction

Ig is a general term for globulins with antibody activity or chemical structure similar to antibodies and produced by B lymphocytes and plasma cells. In recent years, many studies have confirmed that non-B lymphocytes can produce Igs. These cells include a variety of cancer cells such as human salivary gland adenoid cystic carcinoma [1, 2], nasopharyngeal carcinoma [3, 4], laryngeal squamous cell carcinoma [5], parathyroid cancer [6], lung cancer [7–11], gastric cancer [3, 4, 7], liver cancer [7–9, 12–14], pancreatic cancer [5, 9, 15–18], renal clear cell carcinoma [19], cervical cancer [3, 4, 8, 9, 20], ovarian cancer [8, 9], bladder cancer [21], urothelial carcinoma [22], lymphoma [8] and intraductal cancer papillary mucinous tumor [23] and so on. The Igs produced by non-B lymphocytes have similarities and differences with those produced by B lymphocytes and plasma cells. As far as the similarities are concerned, they have the same structure “Y” shape. Their differences are as follows: ⑴ B lymphocyte-derived IgG gene has unlimited diversity, while tumor-derived IgG gene shows limited diversity due to their specific V_H_DJ_H_ recombination patterns and unique mechanisms of somatic hypermutation of functional V_H_ region genes [24–27]. ⑵ B lymphocyte-derived IgG does not contain O-glycans but N-glycans at position Asn297 in the Fc-domain and terminal N-acetylneuraminic acid (NeuAc), while tumor-derived IgG contains not only O-glycans and N-glycans but also NeuAc and N-glycolylneuraminic acid (NeuGc) [28–32]. ⑶ The transcription factors of B lymphocyte-derived IgG include Oct-1, 2, etc., while the transcription factors of tumor-derived IgG has only Oct-1 but not Oct-2 [33, 34]. ⑷ The immunoreactivity of tumor-derived IgG is significantly lower than that of B lymphocyte-derived IgG [35], because the former has abnormal glycosylation modification [36]. ⑸ The functions of B lymphocyte-derived Igs include: ① IgG enhances phagocytosis, neutralizes toxins or viruses, and protects the fetus or neonate; ② IgA protects the mucosa against invasion by microbial pathogens and neonates against microbial infections during the first month of life Infection; ③ IgM prevents microbial pathogens from invading the blood; ④ IgD initiates an immune response; ⑤ IgE plays a crucial role in host resistance to certain parasites and hypersensitivity reactions [37–39]. At present, there are a few reports about the function of tumor-derived Igs, including IgG1, Igα, and Igκ. The functions of the above tumor-derived Igs are as follows: (1) tumor-derived Igs promote tumor cell growth and proliferation [34, 40, 41]; (2) tumor-derived Igs strengthen tumor cell migration, invasion, and metastasis [10, 16]; (3) tumor-derived Igs facilitate tumor immune escape [42–44]; (4) tumor-derived Igs augment drug resistance capacity to chemotherapy drug such as paclitaxel in tumor cells [45]; (5) tumor-derived Igs might regulate the progression of pancreas cancer-associated diabetes [15]; (6) tumor-derived Igs mediate tumor-associated thrombosis by activating platelets after binding to platelet FcγRIIa [46]; (7) tumor-derived Igs maintain cancer stem cell (CSC) potential [45]; (8) tumor-derived IgG may be involved in cell morphogenesis, cell cycle process, fatty acid biosynthetic process, protein biosynthesis, and antimicrobial (virus, bacterium, and fungus) [47]. However, the function of tumor-derived Igλ is still unclear. In this study, we investigated clinical significance and biological functions of tumor-derived Igλ based on bioinformatics data, and then analyzed biological functions of candidate proteins that possibly interact with tumor-derived Igλ after their identification by LC–MS/MS. Our study provided new directions for CESC therapy and functional exploration of tumor-derived Igλ.

## Materials and methods

### Data acquisition and processing

GTEx database (https://www.genome.gov/Funded-Programs-Projects/Genotype-Tissue-Expression-Project, version 2016–09-03) studies the relationship between genetic variation and gene expression in human normal tissues, while TCGA database (https://www.cancer.gov/ccg/research/genome-sequencing/tcga) mainly collects data from cancer tissues. TCGA database was used in combination with GTEx database to analyze the expression levels of 7 IGLCs in 304 CESC tissues and 13 normal tissues. We obtained clinical data of patients and RNA sequencing from the above tumor tissues and normal tissues after log2 (x + 1) transformation. R statistical software package (version 3.6.4) was used to calculate differential gene expression between CESC and normal tissues. In addition, the differences between groups were analyzed by unpaired wilcoxon rank sum test and signed-rank tests. The raw data of 7 IGLC mRNA expressions in the above CESC and normal tissues were in Supplementary Tables 1, 2, 3, 4, 5, 6 and 7. IHC results of cervical adenocarcinoma, cervical squamous cell carcinoma, and corresponding normal cervical tissues were obtained from HPA database (https://www.proteinatlas.org/).

### Analysis of clinical variables

We obtained a series of data including pathological stage (Stage I = 162, II = 69, III = 45, IV = 21), pathological grade (G1 = 18, G2 = 135, G3 = 118), age, T (T1 = 140, T2 = 71, T3 = 20, T4 = 10), N (N0 = 133, N1 = 60), M (M0 = 116, M1 = 10) of patients with CESC from TCGA Pan-Cancer database after log2 (x + 1) transformation. R statistical software package (version 3.6.4) was used to calculate 7 IGLC gene expression differences in different clinical variables of CESC. The differences between groups were analyzed by unpaired Student's *t*-test. The raw data of 7 IGLC mRNA expressions in different clinical variables of CESC, including stage, grade, age, and TNM, were in Supplementary Tables 8–39.

### Prognosis analysis

The outcomes of survival analysis in CESC tissues from TCGA database and their normal tissues from GTEx database, including overall survival (OS), disease-specific survival (DSS), disease-free interval (DFI), and progression-free interval (PFI), were obtained after log2 (x + 1) transformation. Cox proportional hazards regression model constructed by survival package (version 3.2–7) and Kaplan Meier analysis were used to analyze the correlations between 7 IGLC gene expressions and patient prognosis with CESC. The differences between groups were detected by logrank test. The raw data of survival analysis of 7 IGLCs were in Supplementary Tables 40–63.

### Analysis of immunomodulators

The expression data of 5 IGLCs and 150 immunomodulators in CESC tissues from TCGA database and their normal tissues from GTEx database were obtained after log2 (x + 1) transformation. The correlations between IGLCs and immunomodulators were analyzed with a pearson correlation test. The pearson correlation coefficients (r) of 5 IGLCs were in Supplementary Table 64.

### Tumor stemness analysis

Tumor stemness scores of patients with CESC including DNA methylation-based stemness scores (DNAss) and RNA expression-based stemness scores (RNAss) were obtained after log2 (x + 1) transformation. The correlations between tumor stemness scores and 7 IGLC expressions were analyzed with a pearson correlation test. The raw data of DNAss or RNAss and 7 IGLC expressions were in Supplementary Tables 65, 66, 67, 68, 69, 70, 71, 72, 73, 74, 75, 76, 77 and 78.

### Immune cell infiltration analysis

The expression data of 7 IGLCs in CESC tissues from TCGA database and their normal tissues from GTEx database were obtained after log2 (x + 1) transformation. The infiltration scores of tumor-infiltrating immune cells (B cell, T cell CD4, T cell CD8, Neutrophil, Macrophage, and DC) of patients with CESC were obtained by Timer method using R statistical software package (IOBR, version 0.99.9). The correlations between immune cell infiltration scores and 7 IGLC expressions were analyzed with a pearson correlation test using R statistical software package (psych, version 2.1.6). The raw data of immune cell infiltration scores and 7 IGLC expressions were in Supplementary Tables 79, 80, 81, 82, 83, 84 and 85.

### Cell culture and reagents

Human cervical cancer HeLa cells were purchased from BOSTER (Wuhan, Hubei, China). These cells were cultured in Dulbecco’s Modified Eagle’s Medium (DMEM, Sigma-Aldrich, St. Louis, MO, USA) containing 10% fetal bovine serum (FBS, Lonsera, Urguay) and 1% of streptomycin-penicillin Mixtures (Beyotime Biotechnology, Shanghai, China) at 37 ℃ in 5% CO_2_. 10xRIPA lysis buffer was purchased from Merck Millipore (Bedford, MA, USA). Mouse anti-human Igλ antibody and agarose protein G was purchased from Sigma-Aldrich (St. Louis, MO, USA). HRP-conjugated affinipure goat anti-mouse IgG (H + L) secondary antibody was purchased from Proteintech Group, Inc (Hubei, Wuhan, China). Silver staining reagents were purchased from Sangon Biotech (Shanghai, China). Normal mouse IgG were purchased from Zhongshan Golden Bridge Biotechnology (Beijing, China). 3 pairs of cervical cancer tissues and their corresponding normal tissues were obtained from The First Affiliated Hospital of Fujian Medical University.

### Co-IP and western blot

Whole-cell lysates of HeLa cells were incubated with mouse anti-human Igλ antibody or normal mouse IgG at 4 ℃ overnight, and then precipitated with 30 ul protein G agarose for 1 h. After immunocomplexes were washed and boiled, a part of them were separated by sodium dodecyl sulfate polyacrylamide gel electrophoresis (SDS-PAGE) and then detected by silver staining. The other part of immunocomplexes separated by SDS-PAGE were transferred to nitrocellulose membranes. After membranes were blocked with 5% bovine serum albumin, they were incubated with mouse anti-human Igλ antibody at 4 ℃ overnight. The bands were analyzed using Tanon 5200 multi Chemiluminescent Imaging System (Tanon, Shanghai, China) after incubation of HRP- conjugated affinipure goat anti-mouse IgG (H + L) secondary antibodies.

### Silver staining and identification by LC–MS/MS

SDS-PAGE gels were fixed with 40% ethanol and 10% acetic acid for 30 min. Then these gels were sensitized with 6.8% sodium acetate, 0.2% sodium thiosulfate, and 30% ethanol for 30 min. After these gels were washed with deionized water 3 times, they were stained with 0.25% silver nitrate for 20 min and then colored by 2.5% sodium carbonate and 0.0148% formaldehyde respectively. Finally, the above reaction was terminated with 1.46% EDTA for 10 min, and the gels were photographed after washing with deionized water 3 times. The differential bands were cut and sent to Hong Kong Baptist University for LC–MS/MS identification.

### Functional annotation of candidate proteins and bioinformatics analysis

Functional annotation of candidate proteins identified by LC–MS/MS was performed in DAVID (https://david.ncifcrf.gov/). TCGA, GTEx, and GEPIA2 (http://gepia.cancer-pku.cn/) databases were used to analyze bioinformatics of 7 IGLCs in CESC and normal tissues. PPI network analysis was performed on candidate proteins that may interact with tumor-derived Igλ in the STRING database (https://cn.string-db.org/).

### IHC and evaluation of staining intensity for Igλ

IHC was performed as previously described [12]. The mean density of Igλ was quantified using Image-Pro Plus software.

### Statistical analysis

The quantified result of Igλ mean density from IHC was analyzed using SPSS 23.0 software (SPSS, Inc., Chicago, IL, USA) and presented as the mean ± SD. The Student *t*-test was used for the comparison between two groups. Except for the analysis of Igλ mean density, all statistical analyses were performed by the R. *P* < 0.05 was considered statistically significant. Holm-Bonferroni correction was used to correct *P*-values in multiple comparisons.

## Results

### The expression levels of IGLCs between CESC and normal cervical tissues

**Fig. 1:**
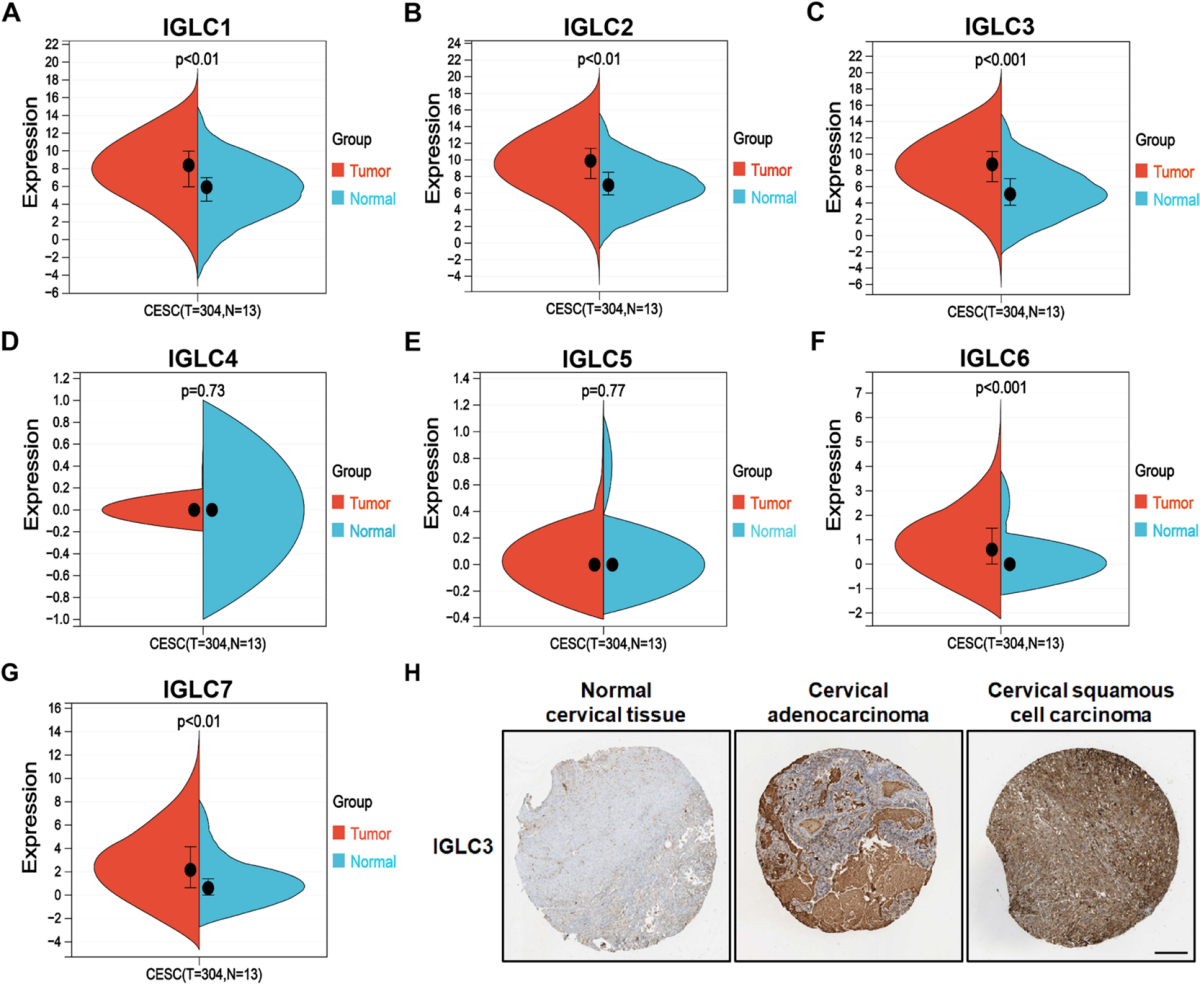
Differences in IGLC expressions between CESC and normal cervical tissues. **A**-**G** Comprehensive analysis of the expressions of IGLC1, IGLC2, IGLC3, IGLC4, IGLC5, IGLC6, and IGLC7 in CESC and normal tissues. *P* < 0.05 was considered statistically significant. **H** Validation of IHC results at the protein level of IGLC3 in normal cervical tissue, cervical adenocarcinoma, and cervical squamous cell carcinoma (scale bar, 200 μm))

The expression levels of 7 IGLCs were analyzed between CESC and normal cervical tissues in TCGA and GTEx database. We found that the expressions of 7 IGLCs (IGLC1, IGLC2, IGLC3, IGLC6, and IGLC7) in CESC tissues were significantly higher than those in normal cervical tissues (Fig. 1A-C, F, G). There was no significant difference in the expressions of IGLC4 and IGLC5 between CESC and normal cervical tissues (Fig. 1D, E). The above results showed that the expressions of the majority of IGLCs were upregulated in CESC tissues. Furthermore, we found that IGLC3 expression level in cervical adenocarcinoma and squamous cell carcinoma was higher than that in normal cervical tissue (Fig. 1H), which confirmed partially the above bioinformatics results of IGLCs.

### The expression levels of IGLCs in different pathologic variables of CESC

**Fig. 2:**
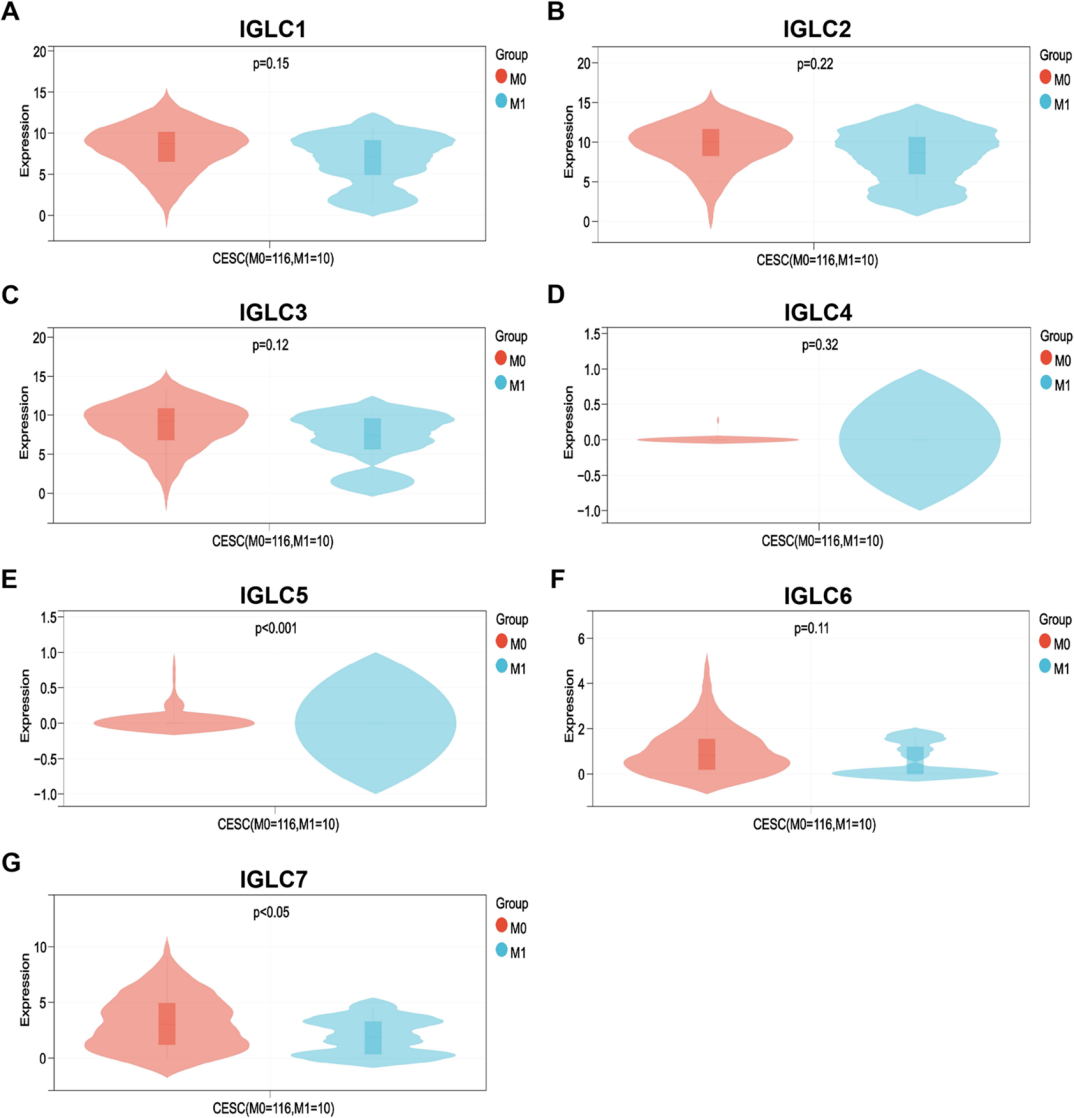
Differences in IGLCs expressions in different pathologic M categories of CESC. **A**-**G** Comprehensive analysis of the expressions of IGLC1, IGLC2, IGLC3, IGLC4, IGLC5, IGLC6, and IGLC7 in two pathologic M categories of CESC. *P* < 0.05 was considered statistically significant

The expressions of 7 IGLCs in different pathological stages, grades, age, and TNM of CESC were analyzed in TCGA database. Our results showed that the expressions of only IGLC5 and IGLC7 among all IGLCs had significant difference in different pathologic M categories of CESC (Fig. 2E, G), while the expressions of IGLC1-4, 6 had no significant difference in different pathologic M categories of CESC (Fig. 2A-D, F). In addition, the expressions of all IGLCs had no significant difference in different pathological stages, grades, age, T, and N of CESC (Supplementary Figs. 1, 2, 3, 4 and 5).

### Prognostic value of IGLCs in CESC patients

**Fig. 3:**
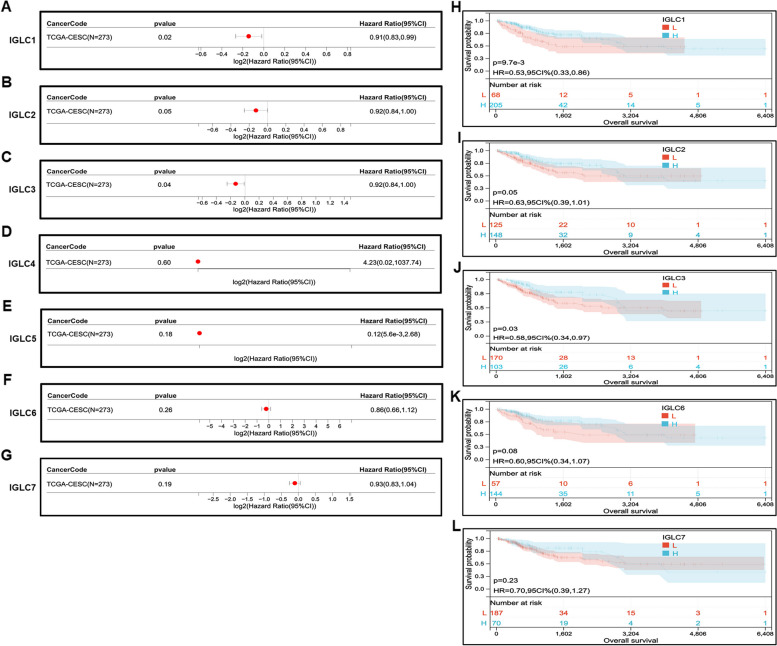
Prognostic assessment of IGLCs expressions from CESC tissues in OS. **A**-**G** Correlation analysis between 7 IGLC (IGLC1, IGLC2, IGLC3, IGLC4, IGLC5, IGLC6, and IGLC7) expressions and OS by utilizing Cox proportional hazards model. **H**–**L** Kaplan–Meier analysis of OS in patients with high and low IGLC expressions. *P* < 0.05 is considered statistically significant. HR > 1 indicates that each of IGLCs may be an adverse factor in the occurrence and development of CESC; 0 < HR < 1 indicates that each of IGLCs may be a protective factor in CESC

The correlations among patient OS, DSS, DFI, PFI and the expressions of 7 IGLCs were analyzed in CESC tissues from TCGA database and their normal tissues from GTEx database. For the correlations between OS and the expressions of 7 IGLCs, our results indicated that the expression levels of IGLC1 (Hazard ratio (HR) = 0.91, *P* = 0.02) and IGLC3 (HR = 0.92, *P* = 0.04) were positively associated with patient OS respectively in CESC according to the Cox proportional hazards model study (Fig. 3A, C). There was no correlation between the expressions of IGLC2 (*P* = 0.05), IGLC4 (*P* = 0.60), IGLC5 (*P* = 0.18), IGLC6 (*P* = 0.26), IGLC7 (*P* = 0.19) and patient OS respectively (Fig. 3B, D-G). Kaplan–Meier analysis showed that low expressions of IGLC1 (*P* = 9.7e-3) and IGLC3 (*P* = 0.03) predicted poor OS respectively in CESC (Fig. 3H, J). There was no correlation between the expressions of IGLC2 (*P* = 0.05), IGLC6 (*P* = 0.08), IGLC7 (*P* = 0.23) and patient OS (Fig. 3I, K, L). For Kaplan–Meier analysis, the cut-off criteria of the L and H groups for IGLG1, 2, 3, 5, 6, and 7 are 6.21, 9.68, 9.56, 0.24, 0.53, and 4.06 respectively. There is no cut-off criteria of the L and H groups for IGLC4. For the correlations between DSS and the expressions of 7 IGLCs, it was indicated that IGLC1 (HR = 0.88,* P* = 8.7e-3), IGLC2 (HR = 0.89, *P* = 0.02), IGLC3 (HR = 0.90, *P* = 0.02) were positively associated with DSS respectively in CESC according to the Cox proportional hazards model study (Supplementary Fig. 6A-C). There was no correlation between the expressions of IGLC4 (*P* = 0.47), IGLC5 (*P* = 0.32), IGLC6 (*P* = 0.07), IGLC7 (*P* = 0.06) and patient DSS respectively (Supplementary Fig. 6D-G). Kaplan–Meier analysis showed that low expressions of IGLC1 (*P* = 4.9e-3), IGLC2 (*P* = 0.01), IGLC3 (*P* = 0.04) and IGLC6 (*P* = 0.04) predicted poor DSS respectively in CESC (Supplementary Fig. 6H-K). IGLC7 expression was not associated with DSS in CESC according to the Cox proportional hazards model study (Supplementary Fig. 6L). For the correlations between DFI and the expressions of 7 IGLCs, it was indicated that all of IGLCs expresses were not associated with DFI respectively in CESC according to the Cox proportional hazards model study (Supplementary Fig. 7A-G). Kaplan–Meier analysis showed that there was no correlation between the expressions of IGLC1 (*P* = 0.09), IGLC2 (*P* = 0.08), IGLC3 (*P* = 0.09), IGLC6 (*P* = 0.05), IGLC7 (*P* = 0.12) and patient DFI respectively (Supplementary Fig. 7H-L). For the correlations between PFI and the expressions of 7 IGLCs, it was indicated that IGLC1 (HR = 0.91, *P* = 0.03), IGLC2 (HR = 0.92, *P* = 0.04), and IGLC7 (HR = 0.88, *P* = 0.02) were positively associated with PFI respectively in CESC according to the Cox proportional hazards model study (Supplementary Fig. 8A, B, G). There was no correlation between the expressions of IGLC3 (*P* = 0.05), IGLC4 (*P* = 0.54), IGLC5 (*P* = 0.23), IGLC6 (*P* = 0.17) and patient PFI respectively (Supplementary Fig. 8C-F). Kaplan–Meier analysis showed that low expressions of IGLC1 (*P* = 0.02), IGLC2 (*P* = 0.04), IGLC3 (*P* = 0.02) and IGLC7 (*P* = 0.02) predicted poor PFI respectively in CESC (Supplementary Fig. 8H-J, L).

### Immunological correlation of IGLCs

**Fig. 4:**
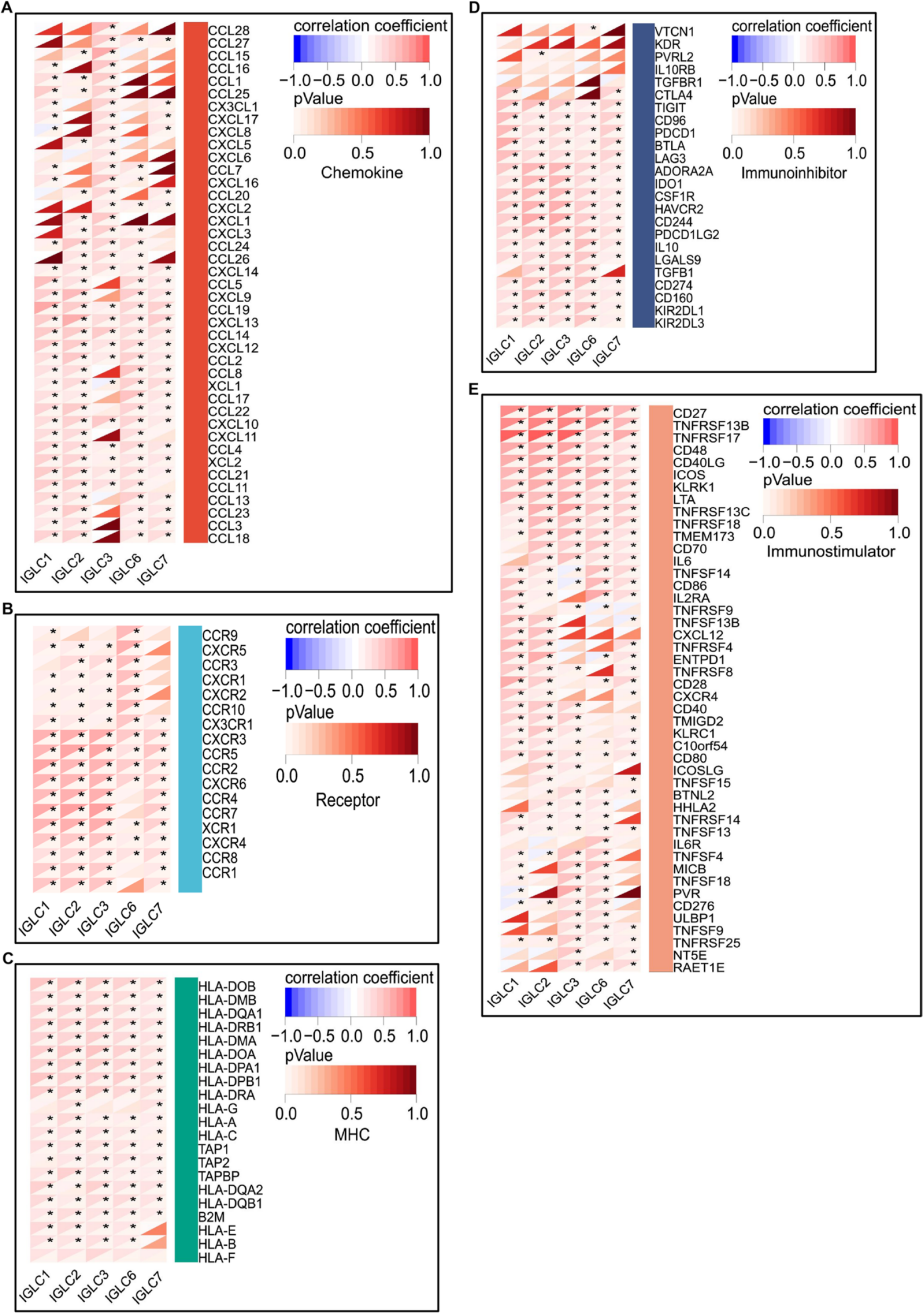
The correlations between immunomodulator expressions and IGLC expressions in CESC tissues. **A**-**E** Determination of the correlations between immunomodulator expressions and the expressions of IGLC1, IGLC2, IGLC3, IGLC6, and IGLC7 in CESC tissues. The color indicates the correlation coefficient or *P*-value. The asterisks indicate a statistically significant *P*-value calculated using pearson correlation analysis. (*P < 0.05)

The correlations between 5 IGLCs (IGLC1-3, 6, 7) and 150 immunomodulators (chemokine (41), receptor (18), MHC (21), immunoinhibitor (24), and immunostimulator (46)) were analyzed in CESC tissues based on TCGA and GTEx databases. Our findings revealed that each of IGLCs was positively correlated with a majority of immunomodulators in CESC tissues (Fig. 4A-E). All of the correlations between IGLCs and immunomodulators were still significant at Bonferroni adjusted *P* < 0.05.

### Tumor stemness and expressions of IGLCs in CESC

**Fig. 5:**
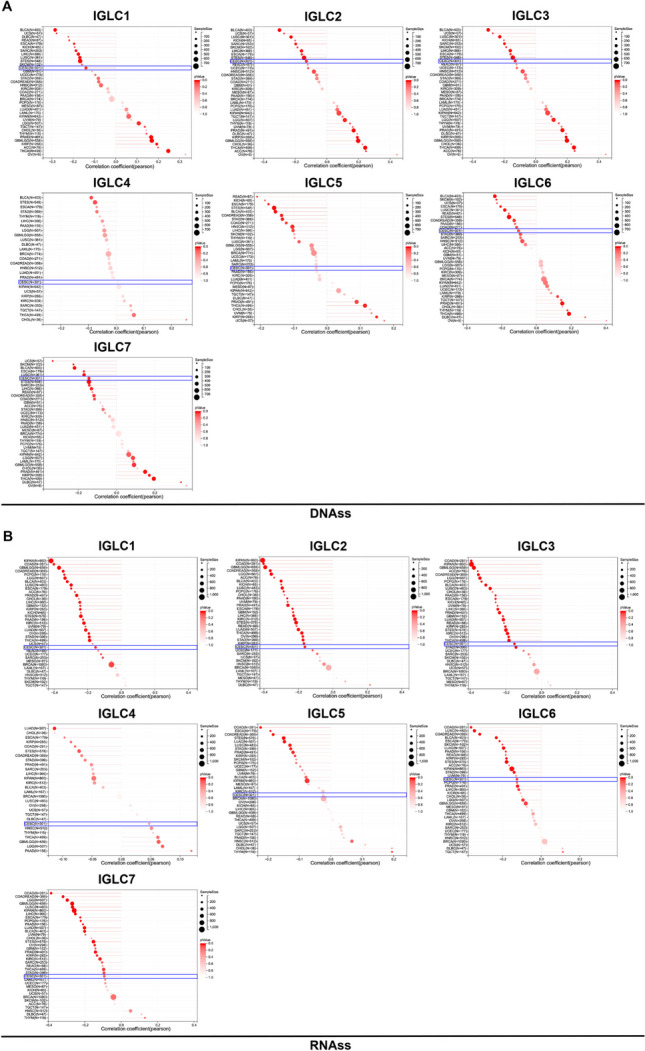
The correlations between tumor stemness and IGLC expressions in CESC tissues. **A**, **B** Correlation analysis between tumor stemness in DNAss and RNAss databases and the expressions of IGLC1, IGLC2, IGLC3, IGLC4, IGLC5, IGLC6, and IGLC7 in 37 types of tumor tissues including CESC marked by blue oblong frame. *r* < 0 and *P* < 0.05 are considered negative correlation. The red color indicates *P*-value, and the deeper the color, the less the *P*-value

The correlations between tumor stemness and the expressions of 7 IGLCs were analyzed in 37 types of tumor tissues including CESC in DNAss and RNAss databases. In DNAss database, we found that tumor stemness was negatively correlated with the expressions of IGLC1 (*r* = -0.14, *P* = 0.02), IGLC2 (*r* = -0.13, *P* = 0.02), IGLC3 (*r* = -0.13, *P* = 0.02), and IGLC7 (*r* = -0.14, *P* = 0.01) respectively in CESC tissues (Fig. 5A). It was found that there was no correlation between tumor stemness and the expressions of 3 IGLCs (IGLC4, IGLC5, and IGLC6) respectively (Fig. 5A). In RNAss database, tumor stemness was confirmed to be negatively correlated with the expressions of IGLC1 (*r* = -0.15, *P* = 0.01), IGLC2 (*r* = -0.16, *P* = 0.01), IGLC3 (*r* = -0.15, *P* = 0.01), and IGLC6 (*r* = -0.13, *P* = 0.03) respectively (Fig. 5B). However, there was no correlation between tumor stemness and the expressions of 3 IGLCs (IGLC4, IGLC5, and IGLC7) respectively (Fig. 5B).

### Immune cell infiltration score and expressions of IGLCs in CESC

**Fig. 6:**
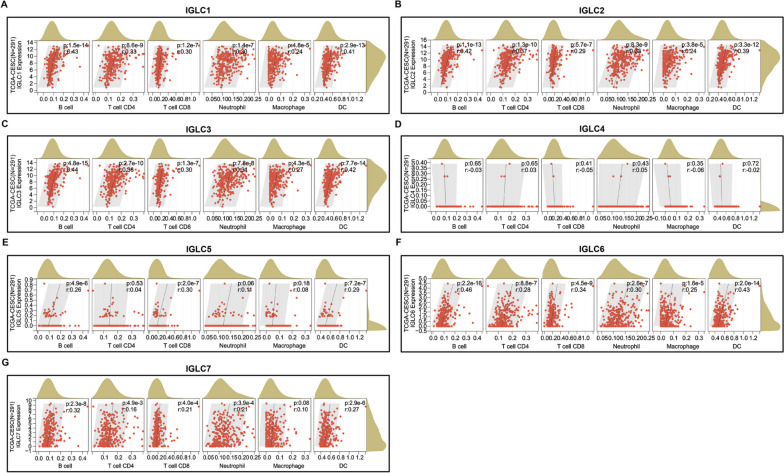
The correlations between immune cell infiltration score and IGLC expressions in CESC. **A**-**G** Correlation analysis between immune cell infiltration score and the expressions of IGLC1, IGLC2, IGLC3, IGLC4, IGLC5, IGLC6 and IGLC7 in CESC tissues. *r* > 0 and* P* < 0.05 are considered positive correlation

The correlations between immune cell infiltration score and the expressions of 7 IGLCs were analyzed in CESC tissues from TCGA database and their normal tissues from GTEx database. It was found that the expressions of 4 IGLCs (IGLC1, IGLC2, IGLC3, and IGLC6) were positively correlated within filtration scores of 6 immune cells respectively, including B cell (*r* = 0.43, *P* < 0.0001; *r* = 0.42, *P* < 0.0001; *r* = 0.44, *P* < 0.0001; *r* = 0.46, *P* < 0.0001), T cell CD4 (*r* = 0.33, *P* < 0.0001; *r* = 0.37, *P* < 0.0001; *r* = 0.36, *P* < 0.0001; *r* = 0.28, *P* < 0.0001), T cell CD8 (*r* = 0.30, *P* < 0.0001; *r* = 0.29, *P* < 0.0001; r = 0.30, *P* < 0.0001; *r* = 0.34, *P* < 0.0001), neutrophil (*r* = 0.30, *P* < 0.0001; *r* = 0.33, *P* < 0.0001; *r* = 0.31, *P* < 0.0001; *r* = 0.30, *P* < 0.0001), macrophage (*r* = 0.24, *P* < 0.0001; *r* = 0.24, *P* < 0.0001; *r* = 0.27, *P* < 0.0001; *r* = 0.25, *P* < 0.001), and DC (*r* = 0.41, *P* < 0.0001; *r* = 0.39, *P* < 0.0001; *r* = 0.42, *P* < 0.0001; *r* = 0.43, *P* < 0.0001) (Fig. 6A-C, F). There was no correlation between immune cell infiltration score and IGLC4 expression (Fig. 6D). For IGLC5, IGLC5 expression was positively correlated with infiltration scores of B cell (*r* = 0.22, *P* < 0.001), T cell CD8 (*r* = 0.26, *P* < 0.0001), and DC (*r* = 0.30, *P* < 0.0001) respectively, while there was no correlation between IGLC5 expression and infiltration scores of T cell CD4, neutrophil, and macrophage respectively (Fig. 6E). For IGLC7, IGLC7 expression was positively correlated with infiltration scores of 5 immune cells respectively, including B cell (*r* = 0.32, *P* < 0.0001), T cell CD4 (*r* = 0.16, *P* < 0.01), T cell CD8 (*r* = 0.21, *P* < 0.001), neutrophil (*r* = 0.21, *P* < 0.001), and DC (*r* = 0.27, *P* < 0.0001), while there was no correlation between IGLC7 expression and infiltration score of macrophage (Fig. 6G). The correlations between immune cell infiltration score and IGLC expressions in CESC remained significant after Holm-Bonferroni correction.

### Silver staining of immune complexes

**Fig. 7:**
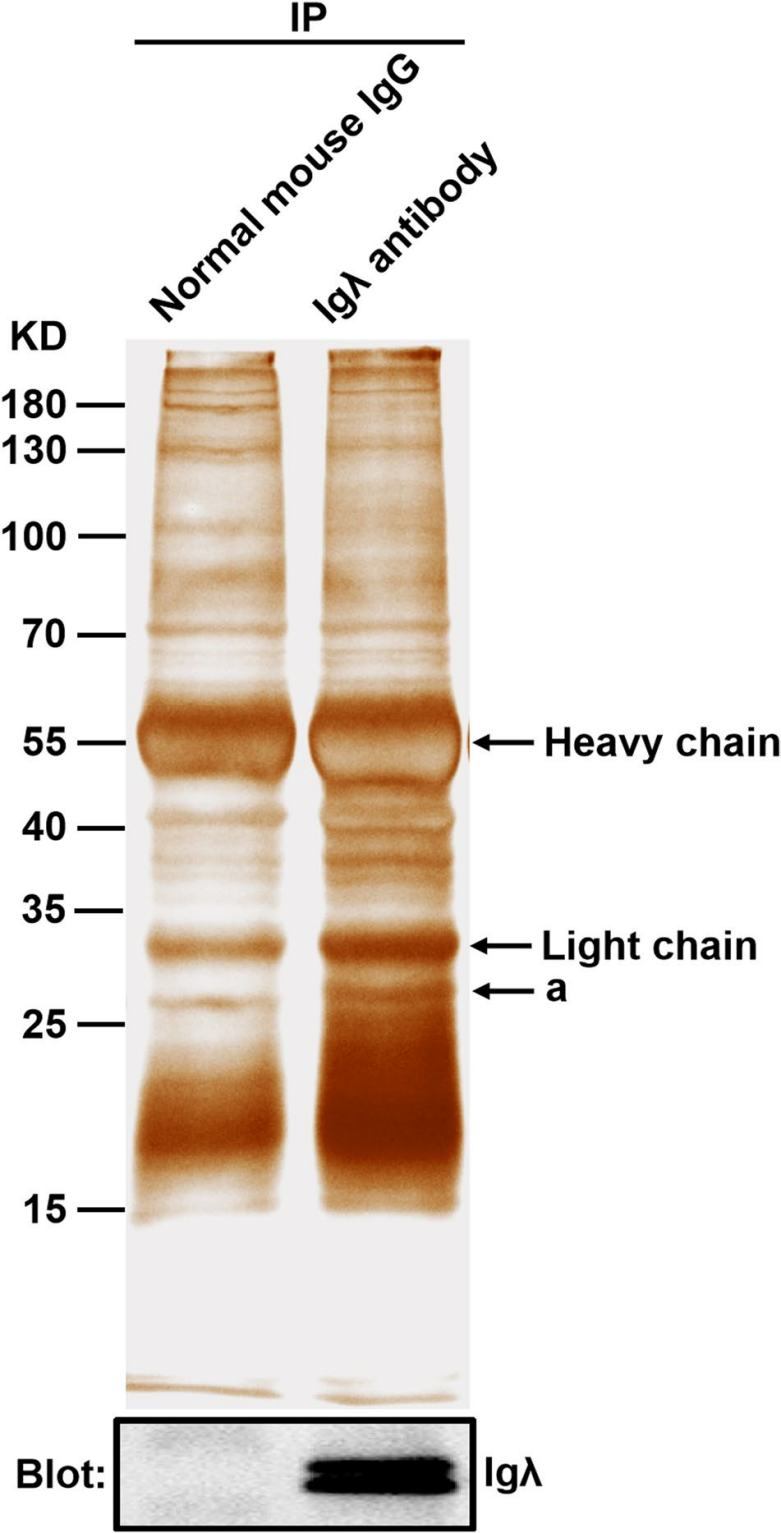
Silver staining of the immunoprecipitate. Proteins immunoprecipitated with normal mouse IgG or mouse anti-human Igλ antibody from the total lysates of HeLa cells were fractionated with 10% SDS-PAGE gel. The gels were visualized with silver staining (upper panel) and blotted with anti-Igλ antibodies (lower panel). The differential band (marked a) was subjected to trypsin digestion and LC–MS/MS analysis

In order to provide the clue for exploring the function of tumor-derived Igλ in cervical carcinogenesis, the immune complexes obtained using Co-IP were separated by SDS-PAGE and then detected with silver staining. One differential band was subjected to protein identification with LC–MS/MS (Fig. 7 marked a). The MS data were analyzed with the Swiss-Prot database. Finally, we identified 4 putative tumor-derived Igλ-associated proteins including ribosomal protein L7 (RPL7), ribosomal protein S3 (RPS3), histone cluster 1, H1b (H1-5), histone cluster 1, H1t (H1-6) (Table 1).

**Table 1:** List of potential tumor-derived Igλ-interacting proteins identified by LC–MS/MS analysis

Accession no	Protein Name	Official Symbol	Mascot score^a^	Queries matched	In-gel digestion^b^
gi|307,388	ribosomal protein L7	RPL7	139	3	a
gi|555,945	ribosomal protein S3	RPS3	94	2	a
gi|75,517,734	histone cluster 1, H1b	H1-5	41	1	a
gi|120,659,986	histone cluster 1, H1t	H1-6	41	1	a

^a^Significance threshold of enumerated proteins was set at *P* < 0.05

^b^a corresponds to those proteins in Fig. 7

### Functional annotation of tumor-derived Igλ-interacting proteins

Four proteins identified by LC–MS/MS were functionally annotated by DAVID. The results showed that these four proteins were involved in various biological processes such as protein biosynthesis, DNA damage and repair, apoptosis, chromosome condensation, Differentiation, and Spermatogenesis (Table 2). Molecular function annotation revealed that RPL7 was a component of the 60S large ribosomal subunit and involved in binding RNA. RPS3, which is a component of the ribosomal 40S subunit and constitutes a part of the translation initiation domain, plays an important role in ribosome biogenesis. H1-5 binds to DNA, RNA, and protein. H1-6, which functions as a developmental protein, is involved in DNA-binding (Table 3). In this study, Co-IP was carried out to obtain four tumor-derived Igλ-interacting proteins. These proteins are distributed in the cytoplasm and nucleus. Annotations from DAVID showed that the above tumor-derived Igλ-interacting proteins were distributed in different parts of cells, suggesting that tumor-derived Igλ performed different functions in different parts of cells.

**Table 2:** Biological process annotations of 4 putative tumor-derived Igλ-binding proteins by DAVID

Candidate proteins	Biological process
RPL7	Protein biosynthesis, rRNA processing
RPS3	DNA damage, DNA repair, Apoptosis, Cell cycle, Cell division, Mitosis, Transcription, Transcription regulation, Translation regulation
H1-5	Chromosome condensation, Nucleosome assembly
H1-6	Differentiation, Spermatogenesis

**Table 3:** Molecular function annotations of 4 putative tumor-derived Igλ-interacting proteins by DAVID

Candidate proteins	Molecular function
RPL7	Ribonucleoprotein, Ribosomal protein, RNA-binding
RPS3	Ribonucleoprotein, Ribosomal protein, Lyase, RNA-binding, DNA-binding
H1-5	DNA-binding
H1-6	Developmental protein, DNA-binding

### Analysis of protein interaction network

**Fig. 8:**
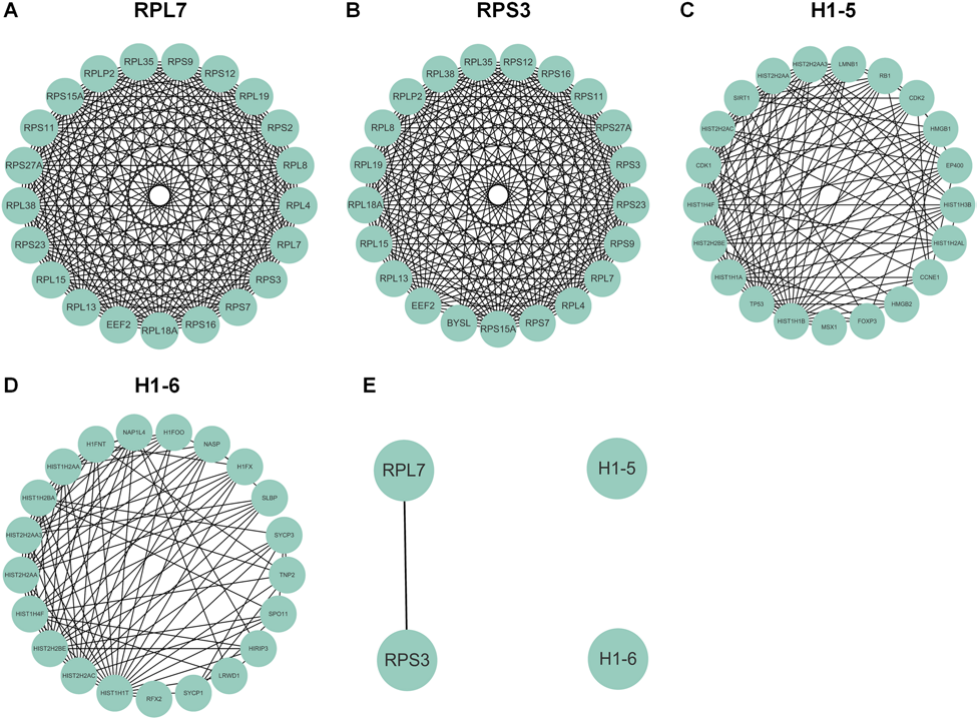
Analysis of tumor-derived Igλ-binding protein interaction network. **A**-**D** PPI network analysis among RPL7, RPS3, H1-5, H1-6, and their interacting proteins. **E** PPI network analysis among RPL7, RPS3, H1-5, and H1-6

Four tumor-derived Igλ-interacting proteins were subjected to single protein PPI network analysis and protein-to-protein interacting network analysis in the STRING database. The obtained data were drawn in Cytoscape 3.9.1 PPI network diagram. According to the above network analysis, RPL7, a tumor-derived Igλ-interacting protein, interacts with the following proteins: RPL35, RPS9, RPS12, RPL19, RPS2, RPL8, RPL4, RPS3, RPS7, RPS16, RPL18A, EEF2, RPL13, RPL15, RPS23, RPL38, RPS27A, RPS11, RPS15A, and RPLP2 (Fig. 8A). RPS3-interacting proteins include RPL35, RPS12, RPS16, RPS11, RPS27A, RPS23, RPS9, RPL7, RPL4, RPS7, RPS15A, BYSL, EEF2, RPL13, RPL15, RPL18A, RPL19, RPL8, RPLP2, and RPL38 (Fig. 8B). H1-5-interacting proteins include LMNB1, RB1, CDK2, HMGB1, EP400, HIST1H3B, HIST1H2AL, CCNE1, HMGB2, FOXP3, MSX1, TP53, HIST1H1A, HIST2H2BE, HIST1H4F, CDK1, HIST2H2AC, SIRT1, HIST2H2AA, and HIST2H2AA3 (Fig. 8C). H1-6-interacting proteins include HIFOO, NASP, HIFX, SLBP, SYCP3, TNP2, SPO11, HIRIP3, LRWD1, SYCP1, RFX2, HIST2H2AC, HIST2H2BE, HIST1H4F, HIST2H2AA, HIST2H2AA3, HIST1H2BA, HIST1H2AA, H1FNT, NAP1L4 (Fig. 8D). In addition, RPL7 directly interacts with RPS3, while H1-5 or H1-6 has no direct interaction with other three tumor-derived Igλ-interacting proteins respectively (Fig. 8E).

### Expression of Igλ in clinical samples

**Fig. 9:**
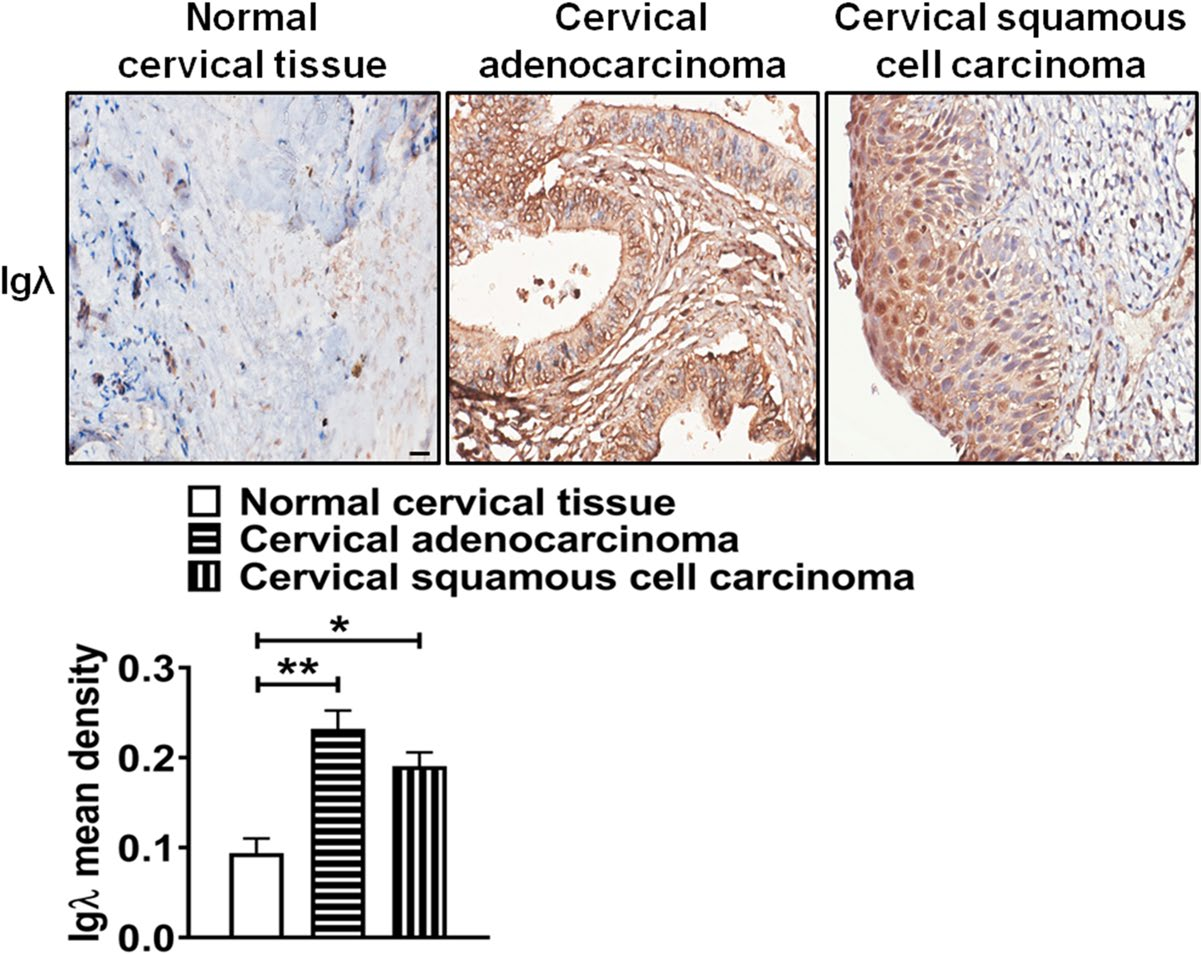
Analysis of Igλ expression in cervical carcinoma tissues and their corresponding normal tissues. Igλ expression (scale bar, 200 μm) in normal cervical tissues, cervical adenocarcinoma tissues, and cervical squamous cell carcinoma tissues. Igλ mean density was analyzed and shown in the lower panel. The data shown are the mean ± S.D. of three independent experiments (*P < 0.05; **P < 0.01)

To further verify our bioinformatics results, we evaluated Igλ expression in normal cervical tissues, cervical adenocarcinoma tissues, and cervical squamous cell carcinoma tissues using IHC. The result showed that the expression level of Igλ in cervical adenocarcinoma and cervical squamous cell carcinoma tissues was higher than that in normal cervical tissues (Fig. 9).

## Discussion

Igs are glycoprotein molecules found in plasma cells that make up an important part of the immune system, which is responsible for fighting off infectious disease and foreign "invasions". There are five classes of Igs in the human body, namely IgA, IgG, IgM, IgE, and IgD. Igs are symmetrical Y-shaped molecules consisting of two longer heavy chains and two shorter light chains. Ig light chains, which ensure the expression and secretion of functional antibodies and contribute to antigen binding, are classified into two types, lambda (λ) and kappa (κ). These chains all interact with each other via either disulfide (S–S) bonds or hydrogen bonds. There are four subclasses of Igλ: Igλ1, Igλ2, Igλ3, and Igλ4 [37, 48, 49]. Many studies have demonstrated that Ig light chains, including Igλ and Igκ, were expressed in a variety of cancer cells. Yang S et al. examined the expressions of Igλ and Igκ in 22 human gastric cancer tissue specimens by the IHC method. The results indicated only Igλ expression in 1 (4.5%), co-expression of Igλ and Igκ in 17 (77.3%), both Igλ and Igκ negative in 2 (9.1%) among all specimens, suggesting that co-expression of Igλ and Igκ in gastric cancer cells was common [50]. Yang SB et al. found that both Igκ and Igλ were not expressed in normal colorectal tissue but in human colorectal cancer cells. Bcl-xL expression was significantly downregulated in HT29 cells after silencing Igλ and/or Igκ, which induced apoptosis of HT29 cells. The result showed that the expression of Igλ and Igκ is necessary to maintain Bcl-xL expression in cancer cells [51]. In addition to gastric cancer cells and colorectal cancer cells, Igλ was also proven to be expressed in other cancer cells, such as cervical cancer [8, 35], hepatocellular cancer [13], breast cancer [8], pancreas cancer [15, 16], prostate cancer [8, 35], lymphoma [52, 53], and acute myelocytic leukemia [54].

RPL7 belongs to the L30P family of ribosomal proteins. As an endonuclease, it has extraribosomal effects and is involved in the repair of UV-induced DNA damage. The interaction of tumor-derived Igλ with RPL7 suggests that tumor-derived Igλ may be involved in DNA damage repair. RPS3, one of ribosomal protein S3 family members, plays a key role in DNA repair, apoptosis, inflammation, tumorigenesis, and transcriptional regulation [55–57]. It was reported that RPS3 enhanced colon cancer cell proliferation, migration, and invasion by decreasing the levels of p53 and lactate dehydrogenase [58]. The interaction of RPS3 protein with tumor-derived Igλ indicates that tumor-derived Igλ may strengthen cancer cell development, including proliferation and metastasis, by impeding p53 signaling pathway. H1-5, a somatic subtype of the histone H1 family, is involved in stabilizing higher-order chromatin structure, regulation of gene expression, DNA repair, cell differentiation, cell proliferation, and cell metastasis [59–61]. The interaction of tumor-derived Igλ with H1-5 demonstrates that tumor-derived Igλ may promote tumor cell proliferation and metastasis. H1-6 is synthesized from the meiotic spermatocyte stage of spermatogenesis [62, 63]. H1-6 is expressed not only in the testis but also in non-germ cells such as cancer cells. H1-6 induces chromatin de-condensation and increases target gene expressions [64]. The interaction of tumor-derived Igλ with H1-6 demonstrates that tumor-derived Igλ may augment chromatin relaxation linked gene activation. In the next step, we will further confirm the interactions and their regions between tumor-derived Igλ and the above 4 proteins using Co-IP, and explore biological functions of tumor-derived Igλ and their action mechanisms. In this research, we demonstrate that higher expressions of IGLCs corresponds to the better OS in Fig. 3. The expressions of the majority of IGLCs in tumor tissues are higher than that in normal tissues in Fig. 1, suggesting that higher expressions of the majority of IGLCs in tumor tissues means the worse OS. The probable reasons for the above paradoxical outcome are as follows: ⑴ Different clinical samples mean different results of analysis because clinical samples used in Fig. 1 are different from those in Fig. 3; ⑵ Tumor tissues have overal higher expressions of IGLCs and all the HRs are lower than 1 in Fig. 3, indicating that higher expression of tumor-derived Igλ corresponds to the better OS. However, if the IGLCs are too highly expressed in tumor tissues, the OS will be worse. In addition, the above results of bioinformatics analysis need to be confirmed by in vitro and in vivo experiments.

In this study, we found that the expressions of the majority of Igλ constants in CESC tissues were higher than normal cervical tissues. The expressions of IGLC5 and IGLC7 had significant difference in two pathologic M categories of CESC. The expression levels of 4 IGLCs were positively associated with patient prognosis respectively in CESC. The expressions of 5 IGLCs were positively correlated with a majority of immunomodulators respectively in CESC. The expressions of 4 IGLCs was negatively correlated with tumor stemness respectively in CESC. Except for IGLC4, IGLC5, and IGLC7, the expressions of 4 IGLCs were positively correlated with infiltration scores of some tumor-infiltrating immune cells respectively. IHC verified IGLC3 expression in CESC and normal cervical tissues. After analysis of a series of bioinformatics data of tumor-derived Igλ, we obtained four potential tumor-derived Igλ-interacting proteins, namely RPL7, RPS3, H1-5, and H1-6, using Co-IP combined with LC–MS/MS. The single protein PPI network and protein-to-protein interacting network of these four proteins were explored. The above proteins are involved in various cellular biological processes, suggesting that tumor-derived Igλ plays a crucial role in tumorigenesis. Furthermore, these proteins also provide valuable clues for studying the functions of tumor-derived Igλ. In summary, we found that Igλ was a novel biomarker for CESC. It had significant correlations with patient prognosis, immunomodulators’ expressions, tumor stemness, and infiltration scores of tumor-infiltrating immune cells in CESC. It expects to be a novel therapy target for CESC.

### Supplementary Information

**Additional file 1:** **Supplementary Table 1.** IGLC1 mRNA expression in CESC and normal tissues.

**Additional file 2:** **Supplementary Table 2.** IGLC2 mRNA expression in CESC and normal tissues.

**Additional file 3:** **Supplementary Table 3.** IGLC3 mRNA expression in CESC and normal tissues.

**Additional file 4:** **Supplementary Table 4.** IGLC4 mRNA expression in CESC and normal tissues.

**Additional file 5:** **Supplementary Table 5.** IGLC5 mRNA expression in CESC and normal tissues.

**Additional file 6:** **Supplementary Table 6.** IGLC6 mRNA expression in CESC and normal tissues.

**Additional file 7:** **Supplementary Table 7.**  IGLC7 mRNA expression in CESC and normal tissues.

**Additional file 8:** **Supplementary Table 8.** IGLC1 mRNA expression in different clinical stages of CESC. **Supplementary Table 9.** IGLC2 mRNA expression in different clinical stages of CESC. **Supplementary Table 10.** IGLC3 mRNA expression in different clinical stages of CESC. **Supplementary Table 11.** IGLC6 mRNA expression in different clinical stages of CESC. **Supplementary Table 12.** IGLC7 mRNA expression in different clinical stages of CESC. **Supplementary Table 13.** IGLC1 mRNA expression in different clinical grades of CESC. **Supplementary Table 14.** IGLC2 mRNA expression in different clinical grades of CESC. **Supplementary Table 15.** IGLC3 mRNA expression in different clinical grades of CESC. **Supplementary Table 16.** IGLC6 mRNA expression in different clinical grades of CESC. **Supplementary Table 17.** IGLC7 mRNA expression in different clinical grades of CESC. **Supplementary Table 18.** IGLC1 mRNA expression in different ages of CESC patients. **Supplementary Table 19.** IGLC2 mRNA expression in different ages of CESC patients. **Supplementary Table 20.** IGLC3 mRNA expression in different ages of CESC patients. **Supplementary Table 21.** IGLC4 mRNA expression in different ages of CESC patients. **Supplementary Table 22.** IGLC5 mRNA expression in different ages of CESC patients. **Supplementary Table 23.** IGLC6 mRNA expression in different ages of CESC patients. **Supplementary Table 24.** IGLC7 mRNA expression in different ages of CESC patients. **Supplementary Table 25.** IGLC1 mRNA expression in different T classifications of CESC. **Supplementary Table 26.** IGLC2 mRNA expression in different T classifications of CESC. **Supplementary Table 27.** IGLC3 mRNA expression in different T classifications of CESC. **Supplementary Table 28.** IGLC6 mRNA expression in different T classifications of CESC. **Supplementary Table 29.** IGLC7 mRNA expression in different T classifications of CESC. **Supplementary Table 30.** IGLC1 mRNA expression in different N classifications of CESC. **Supplementary Table 31.** IGLC2 mRNA expression in different N classifications of CESC. Supplementary Table 32. IGLC3 mRNA expression in different N classifications of CESC. **Supplementary Table 32.** IGLC3 mRNA expression in different N classifications of CESC. **Supplementary Table 33.** IGLC6 mRNA expression in different N classifications of CESC. **Supplementary Table 34.** IGLC7 mRNA expression in different N classifications of CESC. **Supplementary Table 35.** IGLC1 mRNA expression in different M classifications of CESC. **Supplementary Table 36.** IGLC2 mRNA expression in different M classifications of CESC. **Supplementary Table 37.** IGLC3 mRNA expression in different M classifications of CESC. **Supplementary Table 38.** IGLC6 mRNA expression in different M classifications of CESC. **Supplementary Table 39.** IGLC7 mRNA expression in different M classifications of CESC.

**Additional file 9:** **Supplementary Table 40.** OS analysis of patients with CESC. **Supplementary Table 41.** Kaplan Meier analysis of the correlation between IGLC1 gene expression and OS of patients with CESC. **Supplementary Table 42.** Kaplan Meier analysis of the correlation between IGLC2 gene expression and OS of patients with CESC. **Supplementary Table 43.** Kaplan Meier analysis of the correlation between IGLC3 gene expression and OS of patients with CESC. **Supplementary Table 44.** Kaplan Meier analysis of the correlation between IGLC6 gene expression and OS of patients with CESC. **Supplementary Table 45.** Kaplan Meier analysis of the correlation between IGLC7 gene expression and OS of patients with CESC. **Supplementary Table 46.** DSS analysis of patients with CESC. **Supplementary Table 47.** Kaplan Meier analysis of the correlation between IGLC1 gene expression and Dss of patients with CESC. **Supplementary Table 48.** Kaplan Meier analysis of the correlation between IGLC2 gene expression and Dss of patients with CESC. **Supplementary Table 49.** Kaplan Meier analysis of the correlation between IGLC3 gene expression and Dss of patients with CESC. **Supplementary Table 50.** Kaplan Meier analysis of the correlation between IGLC6 gene expression and Dss of patients with CESC. **Supplementary Table 51.** Kaplan Meier analysis of the correlation between IGLC7 gene expression and Dss of patients with CESC. **Supplementary Table 52.** DFI analysis of patients with CESC. **Supplementary Table 53.** Kaplan Meier analysis of the correlation between IGLC1 gene expression and DFI of patients with CESC. **Supplementary Table 54.** Kaplan Meier analysis of the correlation between IGLC2 gene expression and DFI of patients with CESC. **Supplementary Table 55.** Kaplan Meier analysis of the correlation between IGLC3 gene expression and DFI of patients with CESC. **Supplementary Table 56.** Kaplan Meier analysis of the correlation between IGLC6 gene expression and DFI of patients with CESC. **Supplementary Table 57.** Kaplan Meier analysis of the correlation between IGLC7 gene expression and DFI of patients with CESC. **Supplementary Table 58.** PFI analysis of patients with CESC. **Supplementary Table 59.** Kaplan Meier analysis of the correlation between IGLC1 gene expression and PFI of patients with CESC. **Supplementary Table 60.** Kaplan Meier analysis of the correlation between IGLC2 gene expression and PFI of patients with CESC. **Supplementary Table 61.** Kaplan Meier analysis of the correlation between IGLC3 gene expression and PFI of patients with CESC. **Supplementary Table 62.** Kaplan Meier analysis of the correlation between IGLC6 gene expression and PFI of patients with CESC. **Supplementary Table 63.** Kaplan Meier analysis of the correlation between IGLC7 gene expression and PFI of patients with CESC.

**Additional file 10:** **Supplementary Table 64.** The pearson correlation coefficients (r) of 5 IGLCs.

**Additional file 11:** **Supplementary Table 65.** The correlation analysis between DNAss and IGLC1 expression.

**Additional file 12:** **Supplementary Table 66.** The correlation analysis between DNAss and IGLC2 expression.

**Additional file 13: Supplementary Table 67.** The correlation analysis between DNAss and IGLC3 expression. 

**Additional file 14:** **Supplementary Table 68.** The correlation analysis between DNAss and IGLC4 expression.

**Additional file 15:** **Supplementary Table 69.** The correlation analysis between DNAss and IGLC5 expression.

**Additional file 16:** **Supplementary Table 70.** The correlation analysis between DNAss and IGLC6 expression.

**Additional file 17:** **Supplementary Table 71.** The correlation analysis between DNAss and IGLC7 expression.

**Additional file 18:** **Supplementary Table 72.** The correlation analysis between RNAss and IGLC1 expression.

**Additional file 19:** **Supplementary Table 73.** The correlation analysis between RNAss and IGLC2 expression.

**Additional file 20:** **Supplementary Table 74.** The correlation analysis between RNAss and IGLC3 expression.

**Additional file 21:** **Supplementary Table 75.** The correlation analysis between RNAss and IGLC4 expression.

**Additional file 22:** **Supplementary Table 76.** The correlation analysis between RNAss and IGLC5 expression.

**Additional file 23:** **Supplementary Table 77.** The correlation analysis between RNAss and IGLC6 expression.

**Additional file 24:** **Supplementary Table 78.** The correlation analysis between RNAss and IGLC7 expression.

**Additional file 25:** **Supplementary Table 79.** The correlation analysis between immune cell infiltration scores and IGLC1 expression.

**Additional file 26:** **Supplementary Table 80.** The correlation analysis between immune cell infiltration scores and IGLC2 expression.

**Additional file 27:** **Supplementary Table 81.** The correlation analysis between immune cell infiltration scores and IGLC3 expression.

**Additional file 28:** **Supplementary Table 82.** The correlation analysis between immune cell infiltration scores and IGLC4 expression.

**Additional file 29:** **Supplementary Table 83.** The correlation analysis between immune cell infiltration scores and IGLC5 expression.

**Additional file 30:** **Supplementary Table 84.** The correlation analysis between immune cell infiltration scores and IGLC6 expression

**Additional file 31:** **Supplementary Table 85.** The correlation analysis between immune cell infiltration scores and IGLC7 expression.

**Additional file 32:** **Supplementary Fig. 1.** Differences in IGLCs expressions in different patient stage of CESC.

**Additional file 33:** **Supplementary Fig. 2.** Differences in IGLCs expressions in different grade of CESC.

**Additional file 34:** **Supplementary Fig. 3.** Differences in IGLCs expressions in different age of patients with CESC.

**Additional file 35:**  **Supplementary Fig. 4.** Differences in IGLCs expressions in different T of CESC Differences in IGLCs expressions in different T of CESC.

**Additional file 36:** **Supplementary Fig. 5.** Differences in IGLCs expressions in different N of CESC.

**Additional file 37:** **Supplementary Fig. 6.** Prognostic assessment of IGLCs expressions from CESC tissues in DSS.

**Additional file 38:** **Supplementary Fig. 7.** Prognostic assessment of IGLCs expressions from CESC tissues in DFI.

**Additional file 39:** **Supplementary Fig. 8.** Prognostic assessment of IGLCs expressions from CESC tissues in PFI.

**Additional file 40:** **Supplementary Fig. 9.** Raw data for silver staining of the immunoprecipitate.

**Additional file 41:** **Supplementary Fig. 10.** Raw data for western blot result after immunoprecipitation.

**Additional file 42:** **Supplementary Fig. 11.** Raw data for analysis of Igλ expression in cervical carcinoma tissues and their corresponding normal tissues.

## Data Availability

The data used for bioinformatic analysis in this study have already been deposited in TCGA database and GTEx database. The data used for external validation experiments in this study are available from the corresponding authors upon request via email.
