# Supplementary figures and images for: Functional analysis of tumor-derived immunoglobulin lambda and its interacting proteins in cervical cancer
Source: BMC Cancer. 2023 Oct 2;23:929. doi: 10.1186/s12885-023-11426-9 (PMC10544594; doi:10.1186/s12885-023-11426-9)

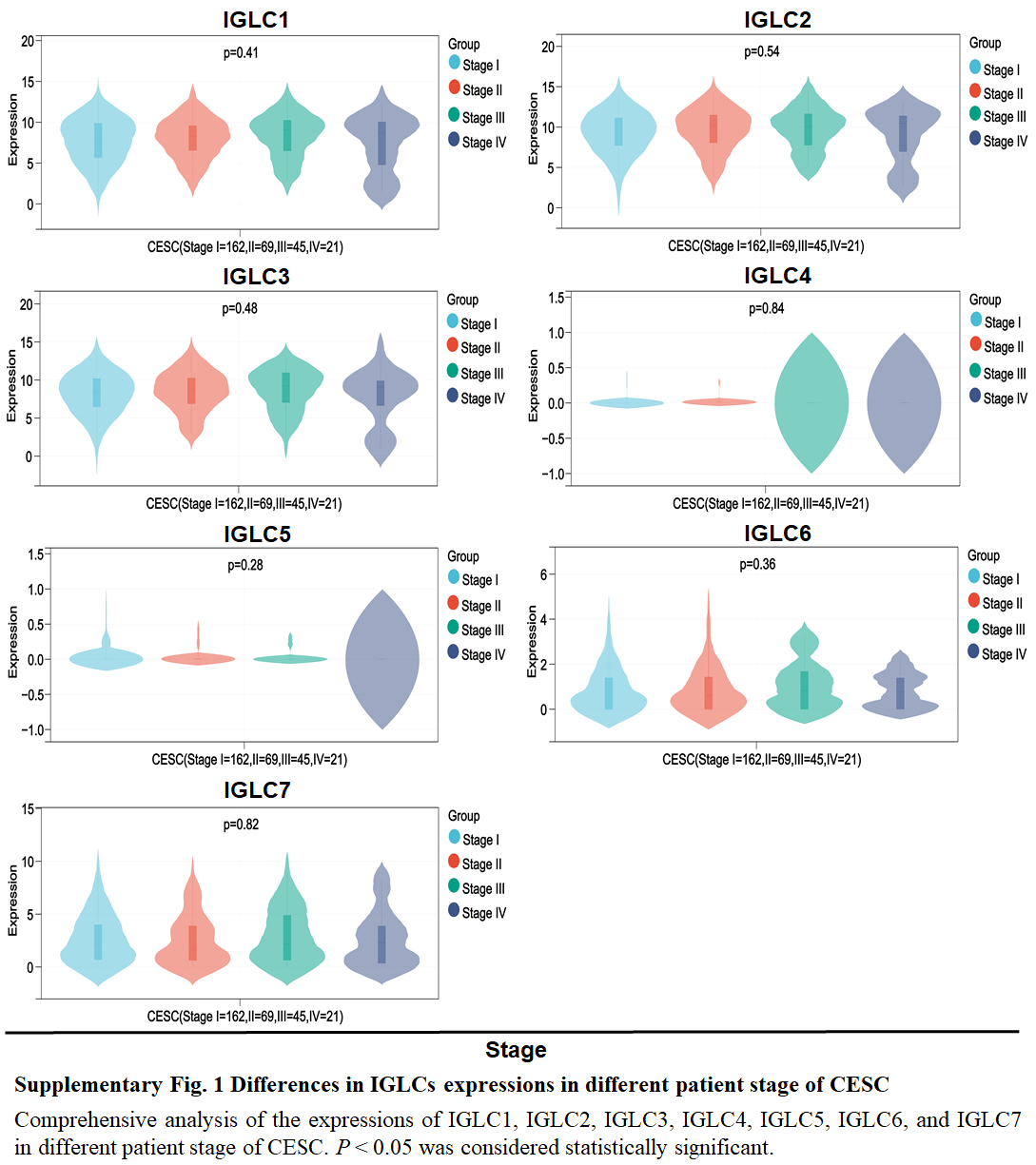

Supplement: Supplementary file 32 — Additional file 32: Supplementary Fig. 1. Differences in IGLCs expressions in different patient stage of CESC. [file 12885_2023_11426_MOESM32_ESM.tif]

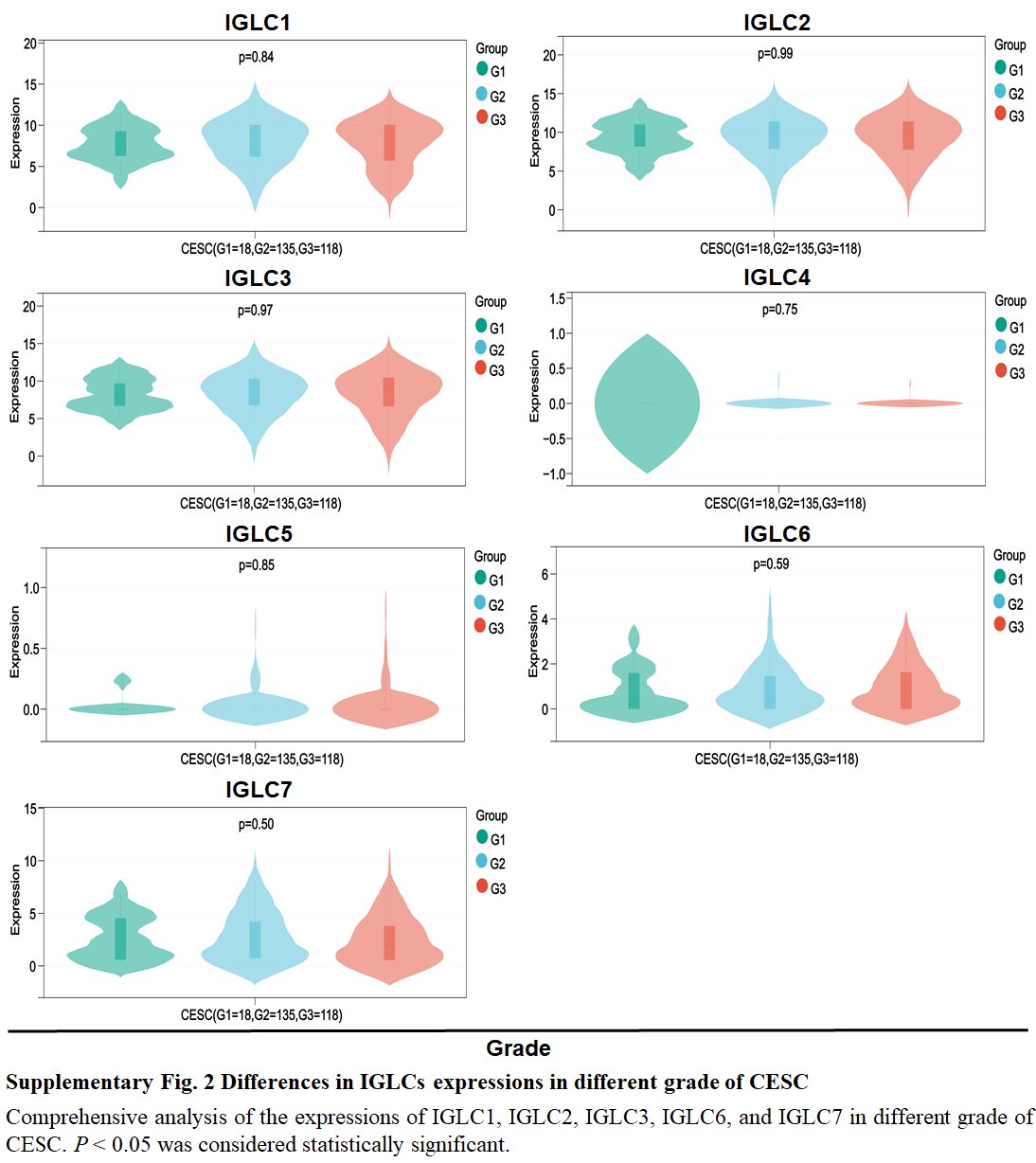

Supplement: Supplementary file 33 — Additional file 33: Supplementary Fig. 2. Differences in IGLCs expressions in different grade of CESC. [file 12885_2023_11426_MOESM33_ESM.tif]

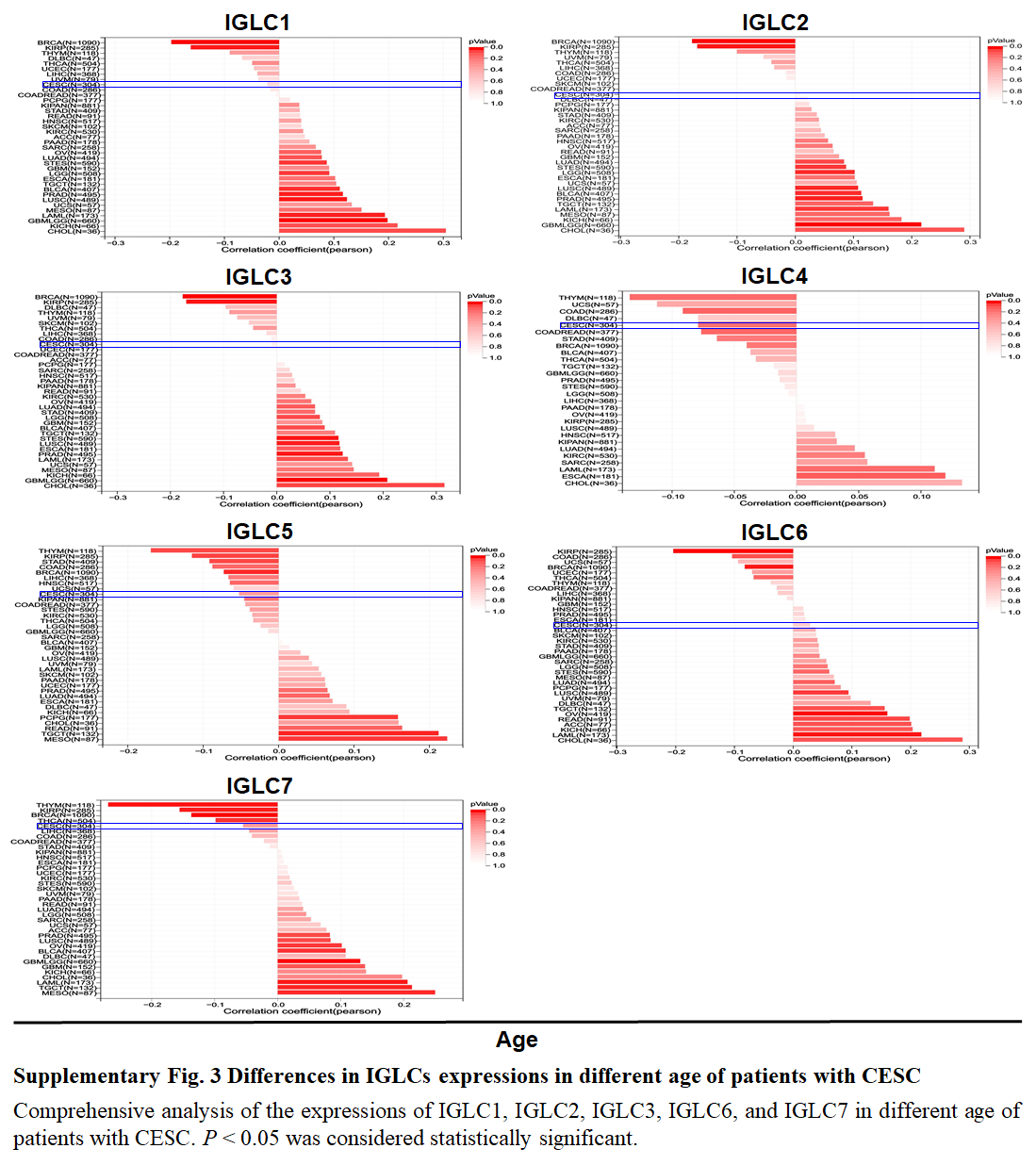

Supplement: Supplementary file 34 — Additional file 34: Supplementary Fig. 3. Differences in IGLCs expressions in different age of patients with CESC. [file 12885_2023_11426_MOESM34_ESM.tif]

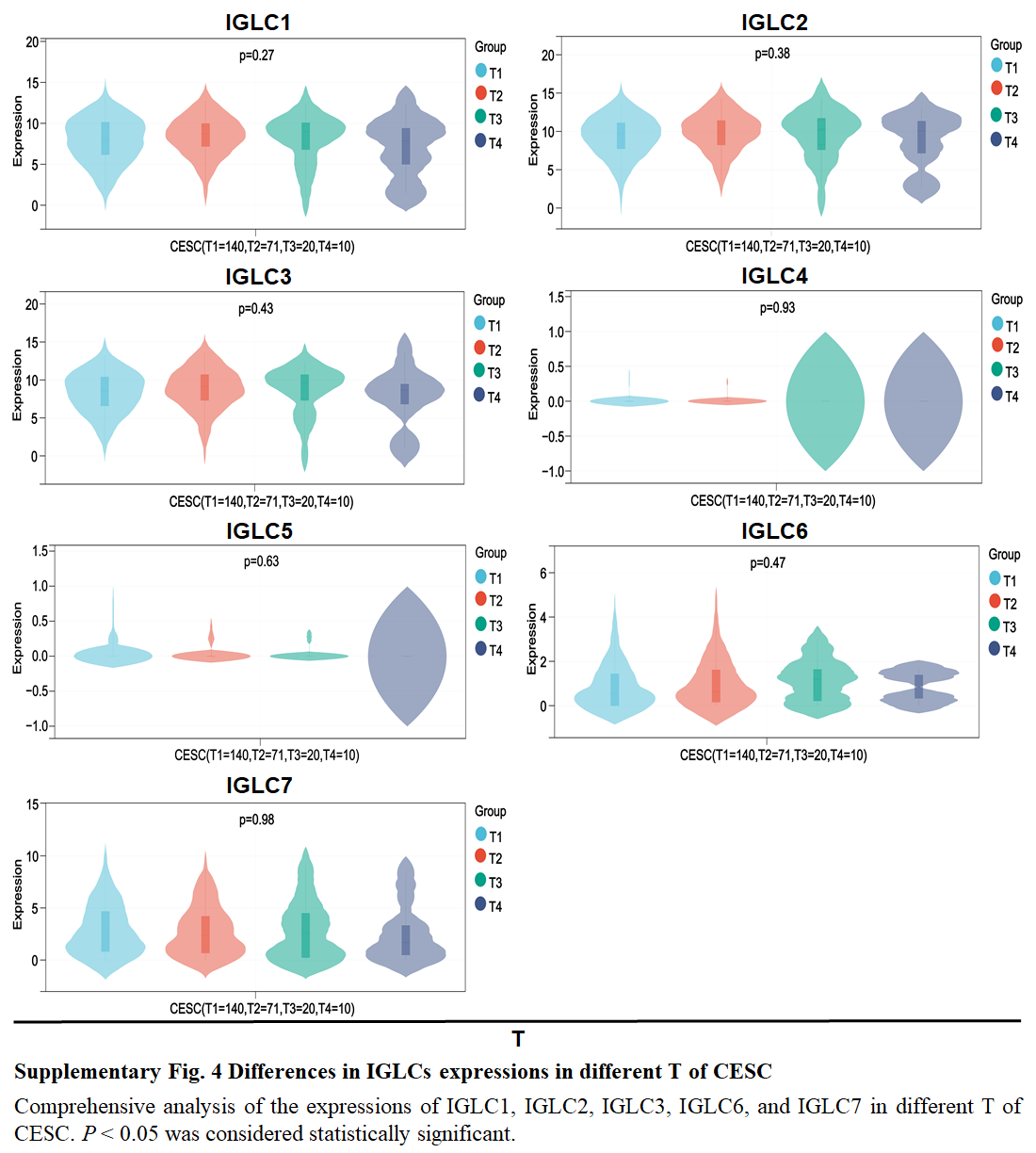

Supplement: Supplementary file 35 — Additional file 35: Supplementary Fig. 4. Differences in IGLCs expressions in different T of CESC Differences in IGLCs expressions in different T of CESC. [file 12885_2023_11426_MOESM35_ESM.tif]

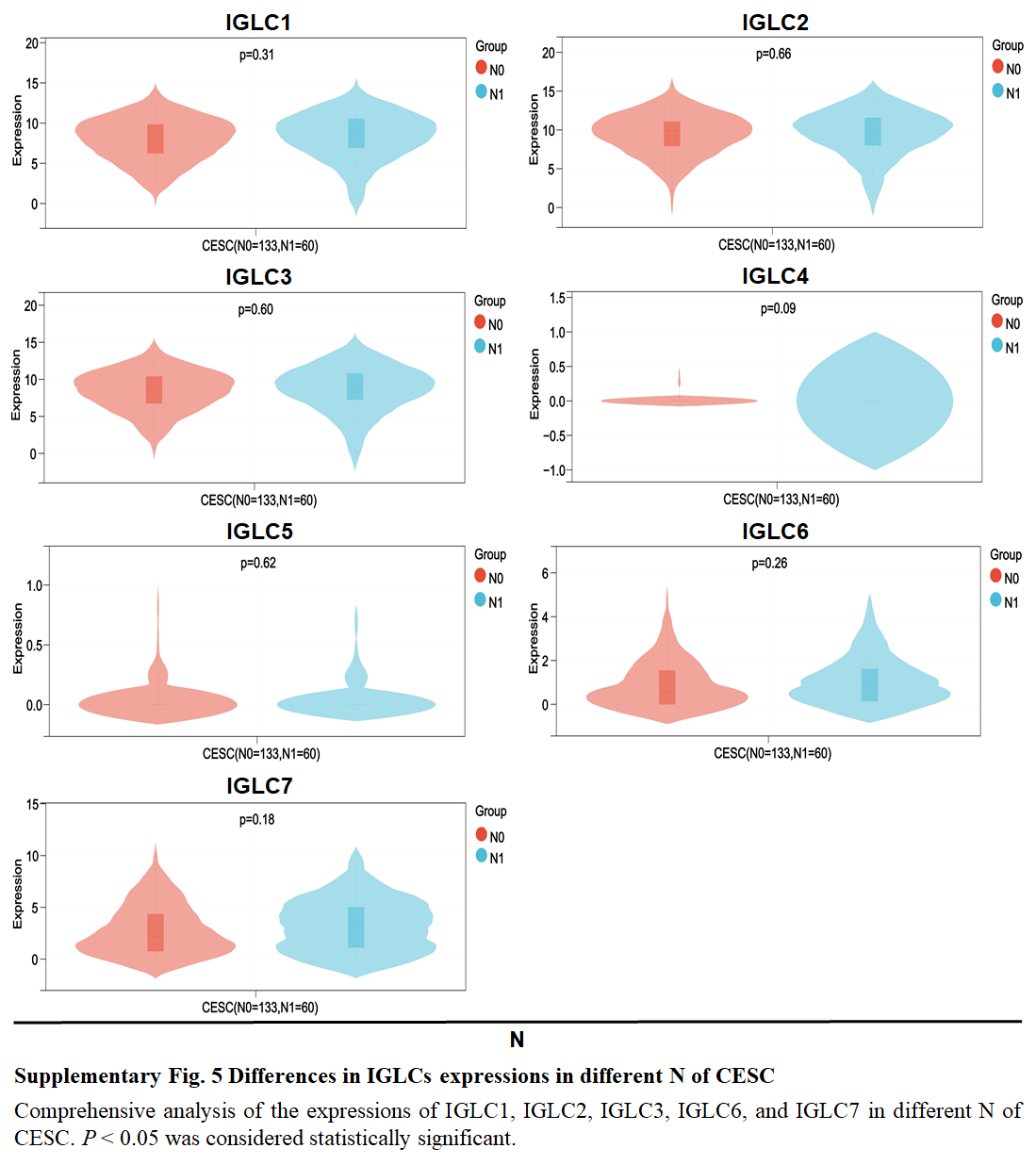

Supplement: Supplementary file 36 — Additional file 36: Supplementary Fig. 5. Differences in IGLCs expressions in different N of CESC. [file 12885_2023_11426_MOESM36_ESM.tif]

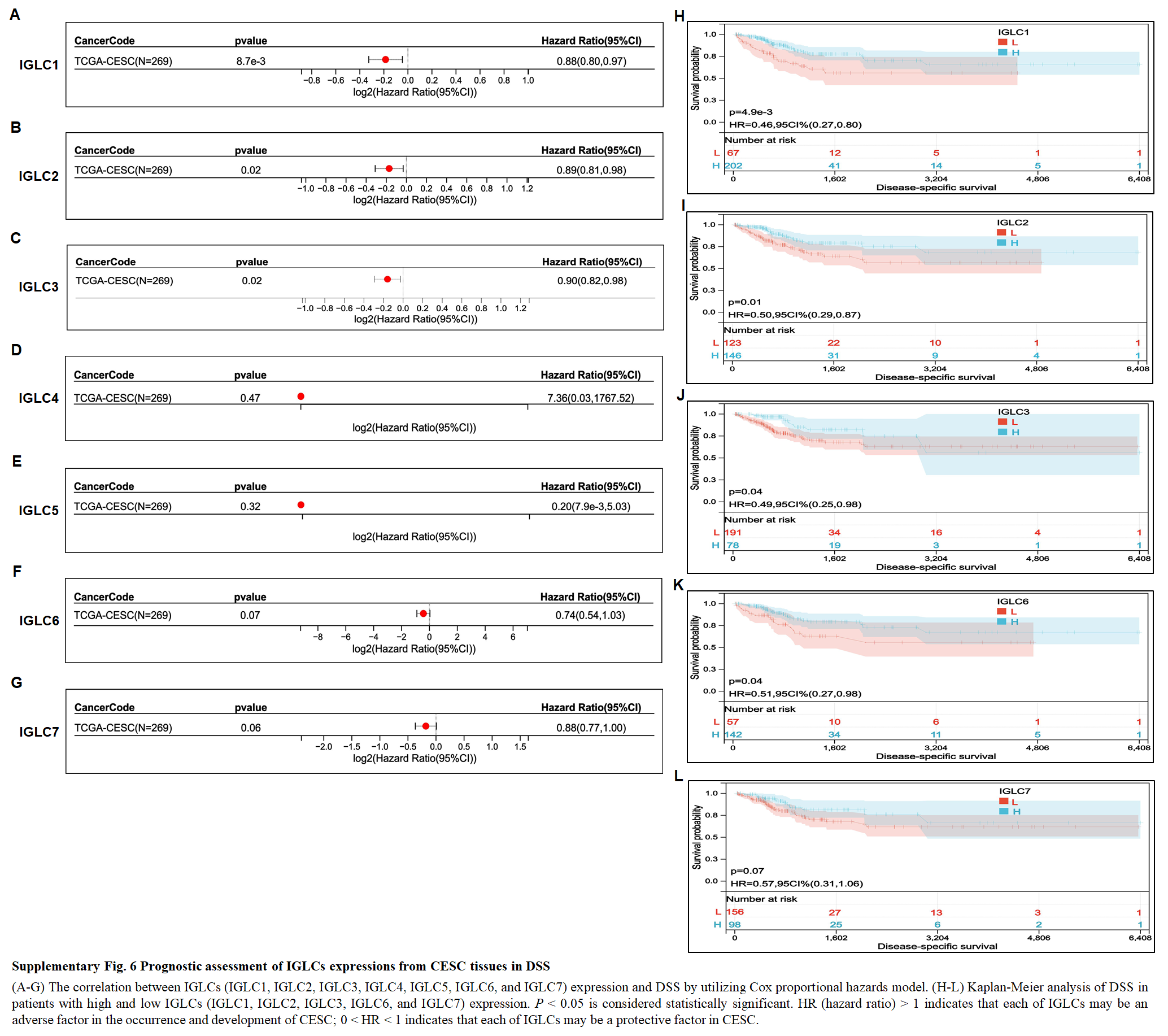

Supplement: Supplementary file 37 — Additional file 37: Supplementary Fig. 6. Prognostic assessment of IGLCs expressions from CESC tissues in DSS. [file 12885_2023_11426_MOESM37_ESM.tif]

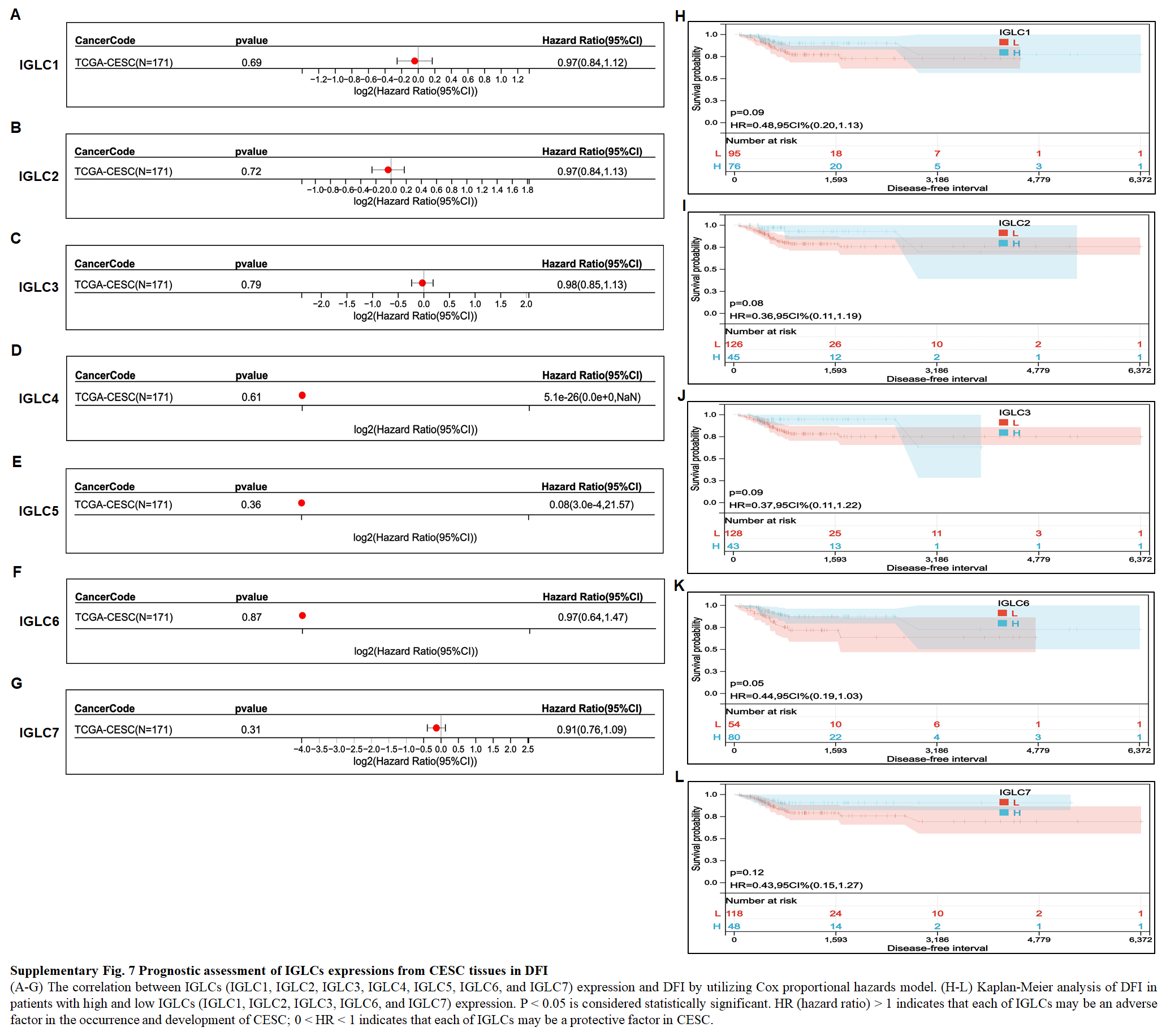

Supplement: Supplementary file 38 — Additional file 38: Supplementary Fig. 7. Prognostic assessment of IGLCs expressions from CESC tissues in DFI. [file 12885_2023_11426_MOESM38_ESM.tif]

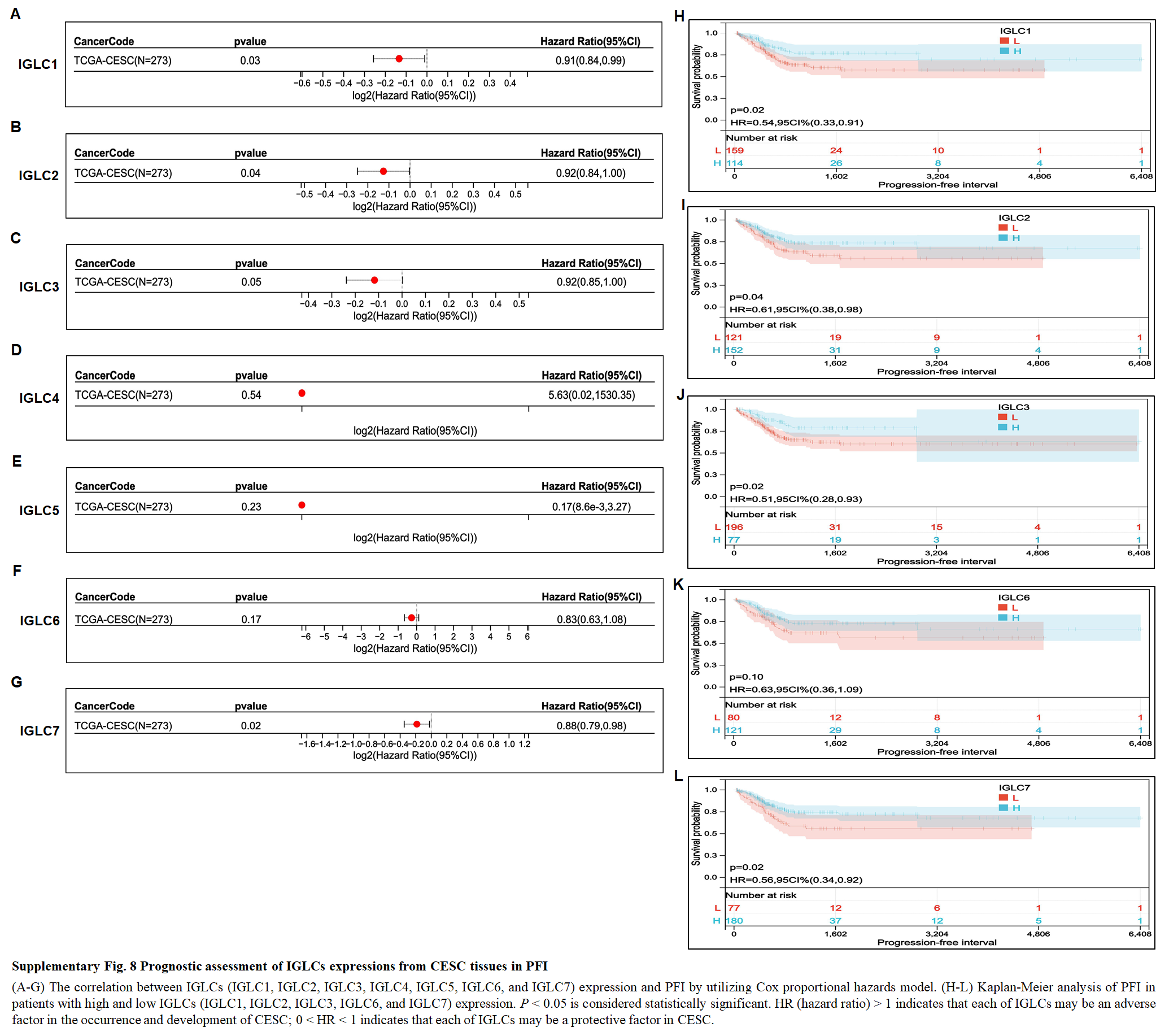

Supplement: Supplementary file 39 — Additional file 39: Supplementary Fig. 8. Prognostic assessment of IGLCs expressions from CESC tissues in PFI. [file 12885_2023_11426_MOESM39_ESM.tif]

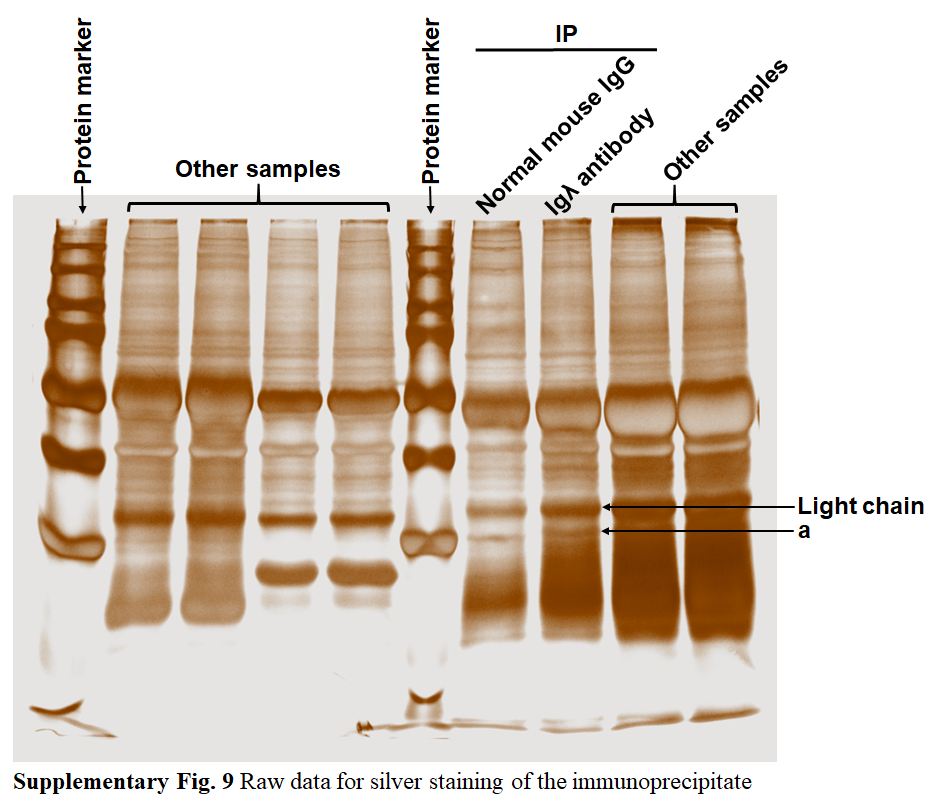

Supplement: Supplementary file 40 — Additional file 40: Supplementary Fig. 9. Raw data for silver staining of the immunoprecipitate. [file 12885_2023_11426_MOESM40_ESM.tif]

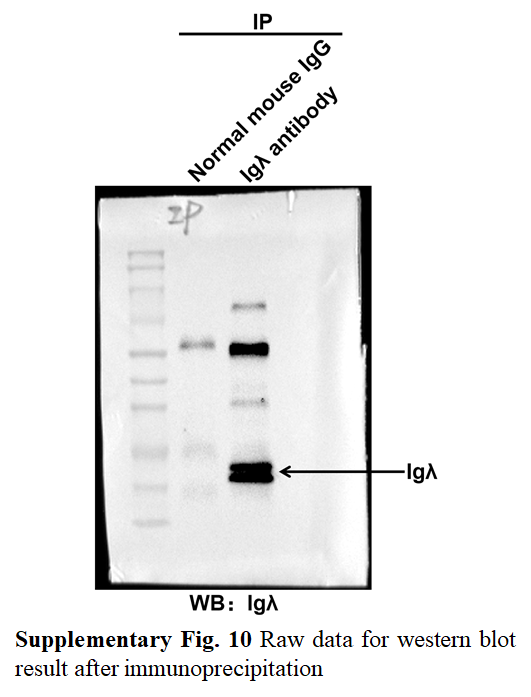

Supplement: Supplementary file 41 — Additional file 41: Supplementary Fig. 10. Raw data for western blot result after immunoprecipitation. [file 12885_2023_11426_MOESM41_ESM.tif]

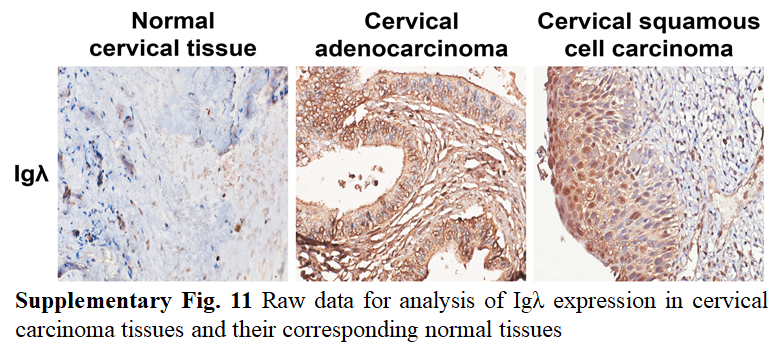

Supplement: Supplementary file 42 — Additional file 42: Supplementary Fig. 11. Raw data for analysis of Igλ expression in cervical carcinoma tissues and their corresponding normal tissues. [file 12885_2023_11426_MOESM42_ESM.tif]
